# Mitochondria at the heart of aging: structure, function, and failure

**DOI:** 10.1186/s12967-026-08047-8

**Published:** 2026-04-24

**Authors:** Hany E. Marei

**Affiliations:** Department of Cytology and Histology, Faculty of Veterinary Medicine, Mansoura University, Mansoura, 35116 Egypt

**Keywords:** Mitochondria, Aging, Mitochondrial dysfunction, Oxidative stress, Mitophagy, Mitochondrial biogenesis, Sirtuins, PGC-1α, NAD+, Rejuvenation

## Abstract

**Background:**

Mitochondria are critical for cellular metabolism, signaling, and health throughout life. Evidence increasingly links age-related mitochondrial dysfunction to a progressive decline in tissue homeostasis. Mitochondrial dysfunction is caused by various mitochondrial DNA (mtDNA) structural and genetic alterations, impaired oxidative phosphorylation, and increased reactive oxygen species (ROS) production. Advances in high-resolution sequencing have revealed vulnerabilities and mutation signatures in tissues previously unknown, reinforcing the precarious balance between mitochondrial damage and injury compensatory response.

**Main body:**

Age-related changes in mitochondrial dynamics, including fission, fusion, and cristae remodeling, are closely related to declining bioenergetic efficiency and cell survival. Disruption of crucial mediators (Drp1, MFN1/2, OPA1) drives cristae degeneration, mimicking pathogenic changes seen in human mitochondrial disorders. Additionally, nutrient and stress sensing pathways, including PGC-1α, AMPK, sirtuins, and mTOR, coordinate mitochondrial biogenesis and metabolic flexibility, linking the energetic status of the organism to the maintenance of organelles. Mitophagy is an important quality-control mechanism that removes damaged mitochondria through the PINK1-Parkin pathway and receptor-mediated pathways involving BNIP3, NIX, and FUNDC1. With aging, this waste-management system deteriorates, compounded by increased ROS and decreased NAD+. Dysregulation of NAD+ metabolism alters the coordination between mitochondrial bioenergetics and signaling. Age-related NAD+ depletion is associated with mitochondrial decline, and preclinical and clinical studies have shown varying, yet moderate medical promise of NAD⁺ precursors including NMN and NR. Several intervention options have emerged, including mitochondria-targeted antioxidants (e.g., MitoQ), mitophagy-activating compounds (e.g., urolithin A), NAD⁺ precursors, senolytics, and gene-based strategies. Despite some progress, challenges remain in establishing reliable biomarkers, developing targeted delivery, and assessing long-term safety. Combating the hindrance of mitochondrial clearance and restorative pathways appears paramount for reinstating organelle function.

**Conclusion:**

Interconnected deficits in genomic stability, dynamics, metabolism, and quality-control systems ultimately cause age-related mitochondrial degeneration. A better understanding of how these approaches into sustained clinical outcomes will require rigorous diagnostic tools and testing interventions. Combining pathways for mitochondrial clearance, degradation, and restoration appears highly promising for countering the effects of aging.

## Introduction

The ageing process gradually leads from molecular, cellular, and systemic functional decline to increased susceptibility to chronic illnesses and diseases. Mitochondrial dysfunction is one of the major biological mechanisms of ageing and has been associated with several age-related conditions such as neurodegenerative disorders, cardiovascular disease and metabolic syndrome [1, 2]. Because mitochondria generate cellular energy, maintain redox balance, and support intracellular signaling, they play a central role in determining both health span and life span.

During the ageing process, mitochondrial genetics, structure and function will exhibit a large amount of remodeling across the various tissues within our bodies. Mitochondrial ageing is characterized by the build-up of mutated and deleted mitochondrial DNA (mtDNA), resulting in decreased efficacy in the process of oxidative phosphorylation, whereby ATP is produced [3]. The simultaneous rise in production of reactive oxygens (ROS) will cause additional oxidative degradation to the structures within the mitochondria as they continue to degrade in functionality.

Most of the information regarding mitochondrial aging has been acquired through research on animal models. Animal model studies have been crucial in developing an understanding of the relationship between mitochondrial (mt) function and different conserved pathways that are involved in the regulation of cellular longevity. Scientists have used various factors (e.g., genetic and environmental) to study the interaction between mitochondria and animal cellular health; however, it remains essential for researchers to distinguish between evidence from animal experiments and that from human studies [4–6].

Human aging has many genetic factors, as opposed to environmental factors and long-term aging. As a result, it will be even more difficult to establish cause-and-effect relationships. For the most part, the evidence regarding human aging comes from: observational studies; evaluations of post-mortem tissue samples; studies of inherited mitochondrial disorders; and recent randomized controlled trials where interventions focus on the mitochondrial pathway, through such methods as the metabolism of NAD⁺ and the provision of energy to cells [7–9]. While a large portion of mitochondrial aging characteristics have comparable counterparts across species, to implement these discoveries derived from computerized species to those of Homo sapiens would require thorough analysis of both the metabolic capabilities and tissue composition of each species. Furthermore, it is critical to appreciate these differences when interpreting research results and designing mitochondria-targeted therapies that will be clinically applicable.

In research performed using newer technologies to conduct many tests at once and examine the DNA from one cell at a time, it has been demonstrated that the mutations in the mtDNA vary depending on the type of body tissue in which they exist, and for the most part, those mutations are not expressed until the need for increased energy output exceeds the ability of the mitochondria to compensate with additional mtDNA replication activities [10]. The data demonstrate that mtDNA pathogenicity can complicate mitochondrial genetic population dynamics, making tissues more prone to the pathogenic effects of naturally occurring (age-related) mtDNA mutations (both latent and clonally expanded) as they begin to present themselves. Additionally, in addition to the genetic instability present in mtDNA, it should be noted that the mitochondria’s capacity to generate energy (bioenergetic competence) and their structural integrity are also modulated by dynamic processes that dictate how these organelles appear and how often they are renewed.

Mitochondrial dynamics, which encompass processes such as fission, fusion, and the remodeling of cristae, play a crucial role in sustaining proper function and adapting to metabolic stress. The age-related imbalances in these processes promote increased fragmentation, altered cristae structure and diminished ability to efficiently respire. The protein mis regulation of factors that regulate mitochondrial architecture has a major effect on the age-related dis-functioning and illness of mitochondria [11, 12]. These structural abnormalities further sensitize the mitochondria to damage and reduce their capacity for functional renewal.

Cells employ quality control mechanisms to preserve the integrity of their mitochondria, with a prominent quality control mechanism being mitophagy. Mitochondrial dysfunction (e.g., damaged) is removed via the PINK1-Parkin pathway through selective removal and a variety of receptor-mediated pathways (e.g., BNIP3, NIX, FUNDC1) to support the preservation of cell homeostasis. However, as we get older, there is a decrease in the efficiency of mitophagy, which ultimately results in an accumulation of dysfunctional organelles, a diminished ability to switch between different metabolic pathways, and an increase in oxidative stress [13]. The decline in mitophagic efficiency coincides with changes to nutrient- and stress-sensing pathways that regulate both mitochondrial biogenesis and turnover as we age.

Central to all of these pathways are the NAD⁺, which is a co-factor critical to maintaining redox balance, mitochondrial metabolism and longevity-associated signaling pathways. As aging progresses, NAD+ (Nicotinamide adenine dinucleotide) levels are decrease, hence sirtuins, AMP-activated protein kinase (AMPK) and PGC-1 alpha activity is decreased but mTOR (mechanistic target of rapamycin) signaling is dysfunctional affected [14]. The use of NMN and NR has demonstrated beneficial effects on muscle; however, clinical results to date have demonstrated a mixed and context-dependent response to supplementation with these metabolites [15].

Mitochondria are not simply passive targets of the ageing process; they are actively involved with defining an organism’s longevity and health. This new understanding has created significant interest in the potential use of mitochondrial-targeting therapies to enhance longevity by restoring mitochondrial function/supply of NAD⁺; mitochondrial-type antioxidants, such as MitoQ; mitophagic-enhancing agents, such as urolithin A; senolytic therapies, and genetic engineering/reprogramming of mitochondrial pathways and function [16, 17]. Additionally, despite the progress being made with respect to mitochondrial-targeted therapies, significant barriers exist, such as developing reliable biomarkers, developing targeted delivery systems and determining the long-term safety and effectiveness of these therapies.

Recent research indicates that declining ability to adjust to changes in the environment (i.e., mitochondrial function) is an indication of a lack of ability to adapt, rather than just damage accumulating over time: (a) detection of damage, (b) quality control of damaged components, and (c) bioenergetic remodeling to meet increased demand [1, 2, 10]. In this model, mtDNA instability, altered dynamics of mtDNA, decreased ability to efficiently remove damaged mitochondria through mitophagy, and the disruption of metabolic signaling are not completely separate; rather, they function together as part of a larger regulatory network, and their coordination decreases with age. Many of the changes to mitochondria observed with age may represent an initial compensatory response that becomes maladaptive when engaged over a prolonged period or inadequately resolved [18, 19]. The model outlined above reconciles some of the conflicting results reported in the literature regarding the effects of reactive oxygen species, mitochondrial fusion, and mitophagy. Additionally, this model clearly distinguishes the primary causes of mitochondrial dysfunction from its secondary effects and failed adaptations. Understanding mitochondrial aging as a broadening, system-wide loss of adaptability underscores the criticality of therapies that restore the overall mitochondrial environment to a state of homeostasis, rather than focusing on one or two distinct pathways in need of restoration.

This review discusses a conceptual framework that views mitochondria as a central regulatory hub and an integration point for cellular pathways involved in genetic, metabolic, and quality-control functions. Under this model, mitochondria coordinate dynamic changes in mtDNA, metabolic signaling, including NAD+, and mitophagy, thereby preserving cellular resilience through coordinated adaptation. The ageing process does not represent a failure of a single process, but rather a progressive reduction of the ability of mitochondria to process information and adapt to environmental changes from an integrated network of pathways. The review will also focus on mtDNA disturbances; the dynamics of mitochondrial function and mitophagy; the role of signaling pathways involved in mitochondrial biogenesis and mitophagy, and their ability to restore or enhance mitochondrial health; and, lastly, the potential use of these therapies as novel anti-ageing strategies.

## Foundational and conceptual frameworks of aging

Aging is a universal biological process marked by a decline in cellular and organismal function, increasing the risk of disease and death. Research into the causes of aging is ongoing, but the process itself cannot be stopped. The complex process of aging involves many cellular and molecular pathways, many of which are also linked to age-related diseases. Over the last 20 years, ideas such as the “hallmarks of aging” have provided a structured framework for studying aging [1]. At the same time, mitochondria, which are essential organelles that help with energy metabolism, signaling, and apoptosis, have become important in both normal and disease-related aging [2, 3]. New research is helping us understand how mitochondrial function, oxidative stress, inflammation, and systemic aging all work together [4, 5].

### The hallmarks of aging as a framework

Lopez-Otin and his team did a groundbreaking review in which they grouped the aging process into nine related traits. Genomic instability, telomere attrition, epigenetic modifications, proteostasis loss, dysregulated nutrient sensing, mitochondrial dysfunction, cellular senescence, stem cell depletion, and altered intercellular communication are among them. This field demonstrated the interrelation of these characteristics through a cohesive theoretical framework. Genomic instability is a sign of mitochondrial dysfunction. It happens when there are more reactive oxygen species (ROS), when metabolism fails, which speeds up aging, and when mitochondrial damage-associated molecular patterns (DAMPs) are released [1].

The hallmarks framework helps learn how to communicate with people. Caloric restriction, NAD+ boosters, and mitochondrial-targeted antioxidants are examples of therapies that may be linked to one or more hallmarks. We can now study how aging systematically affects biology.

### Mitochondria as central players in aging

Mitochondria not only provide the cell with energy, but also help it stay alive by monitoring and controlling how much energy it uses. The free radical theory, which posits that oxidative phosphorylation generates reactive oxygen species (ROS) that accumulate and damage cellular components, was an early study that highlighted the significance of ROS in aging. There have been advancements to this theory since its inception; however, it is still posited that bioenergetic failure, reactive oxygen species (ROS) imbalance, and metabolic signaling converge in mitochondria to induce aging [1, 20].

**Fig. 1:**
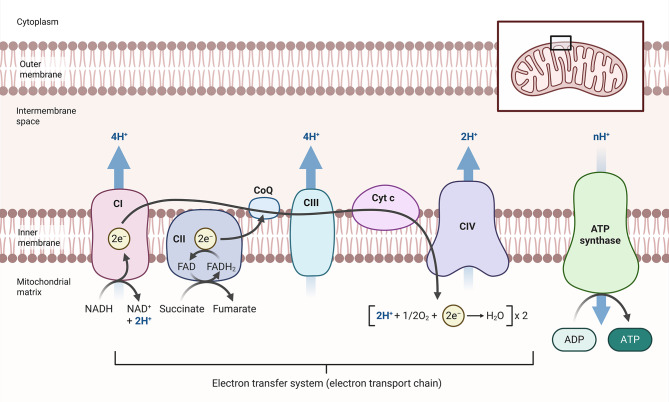
An overview of mitochondria and their connection to aging. The electron transport chain (ETC) complexes, cristae, and mitochondrial DNA (mtDNA) are at the center of this mitochondrial diagram. The “spokes” encircling the perimeter represent significant aging-related events, such as changes in dynamics (fission/fusion), a decrease in mitophagy, a depletion of NAD⁺, and connections to cellular senescence and inflammaging

Bratic and Larsson demonstrated that mitochondrial dysfunction not only serves as a marker of aging but can also accelerate the aging process. Mouse models exhibiting deficient mitochondrial DNA (mtDNA) replication demonstrated a causal relationship between mitochondrial integrity and organismal lifespan. These mice gather mutations and show signs of early aging. Similarly, treatments that maintain mitochondrial function often prolong animal lifespan. Mitochondria are not only markers of aging, but they also accelerate it (Fig. 1).

### Expanding the mitochondrial paradigm: beyond energy production

More modern ideas, such as Srivastava3’s, have expanded the mitochondrial paradigm by linking mitochondrial dysfunction to specific age-related diseases, including metabolic syndrome, cardiovascular disease, and dementia. Srivastava noted that signs of mitochondrial decline include reduced ATP production, disturbances in mitochondrial dynamics (fusion/fission), defects in mitophagy, and abnormal ROS signaling. These problems feed on each other, accelerating the system’s death [2, 21].

Mitochondrial failure is a significant part of aging and interacts with other signs of aging. For instance, when mTOR or AMPK signaling isn’t functioning correctly, it affects mitochondrial production and breakdown. When proteostasis isn’t working properly, the structure of the mitochondrial proteome is disrupted. Mitochondria are an essential part of aging because they bring together many signs of aging into one picture [1, 22].

### Mitochondria, oxidative stress, and inflammation

Xu et al. demonstrate that mitochondria facilitate aging by generating excessive reactive oxygen species (ROS) and, crucially, by participating in immune signaling. Redox stress and inflammation are elements of “inflammaging.” Recent research indicates that mitochondria play a role in both processes. Mitochondrial damage activates innate immune pathways, leading to chronic inflammation by releasing mitochondrial DNA and other DNA-associated molecular patterns (DAMPs) into the extracellular space or the cytoplasm [23].

This mitochondrial-inflammatory axis not only accelerates tissue breakdown but also further harms mitochondria by prolonging the inflammatory response. The authors suggest that new ways to slow down or even reverse aging may involve preventing mitochondria from producing damage-associated molecular patterns (DAMPs) or reactive oxygen species (ROS). This idea goes beyond the organelle-centered view of the last few decades and puts mitochondria in the bigger picture of how the body and the system age [24, 25].

### Mitochondrial dysfunction as a systemic driver of aging

Zhang et al. enhance the theoretical framework by illustrating that mitochondrial failure constitutes a systemic, rather than a cell-specific, etiology of aging. Their research indicates that mitochondrial dysfunction affects numerous processes, including tissue-specific metabolism, intercellular signaling, and stem cell maintenance. When mitochondria detect an anomaly, they signal other organelles in the body via messenger vesicles and metabolites [26].

This systemic perspective aligns with the notion that aging is characterized by an incremental, systemic deterioration of the entire body’s systems, associated with organelle-level dysfunction that is disseminated through signaling networks. Effective therapeutic strategies to ameliorate age-related diseases should focus on restoring metabolic integration and reestablishing systemic communication [26].

### Integrative perspectives and implications

The hallmarks framework and the essential function of mitochondria are the primary concepts underlying research on aging. Lopez-Otin et al. [1] laid the groundwork, and Bratic and Larsson2 emphasized the indispensable role of mitochondria. Further investigations by Xu et al. [23] and Zhang et al. [26] expanded the focus to include the role of mitochondria in inflammation, systemic dysfunction, and inter-organ communication; Srivastava elucidated a link between these findings and human disease.

We learn more about how things work, and these models help translational science. For instance, these theoretical models can be utilized to evaluate the efficacy of NAD⁺ boosters. These antioxidants specifically target mitochondria, such as MitoQ, or mitophagy activators like urolithin A, simultaneously reducing multiple characteristics [27, 28].

Finally, the link between mitochondria and aging is emerging as a focus of the growing body of research stemming from multiple theories. At the molecular and systemic levels, research on mitochondria has elucidated that these organelles contribute to the aging process [21, 23, 29] Continued elucidation of these models provides a framework for advancing methodological approaches, therapeutic studies, and translational research. Improving healthy lifespan and slowing the effects of aging are closely associated with early monitoring of hallmark-based methods in mitochondrial biology.

**Fig. 2:**
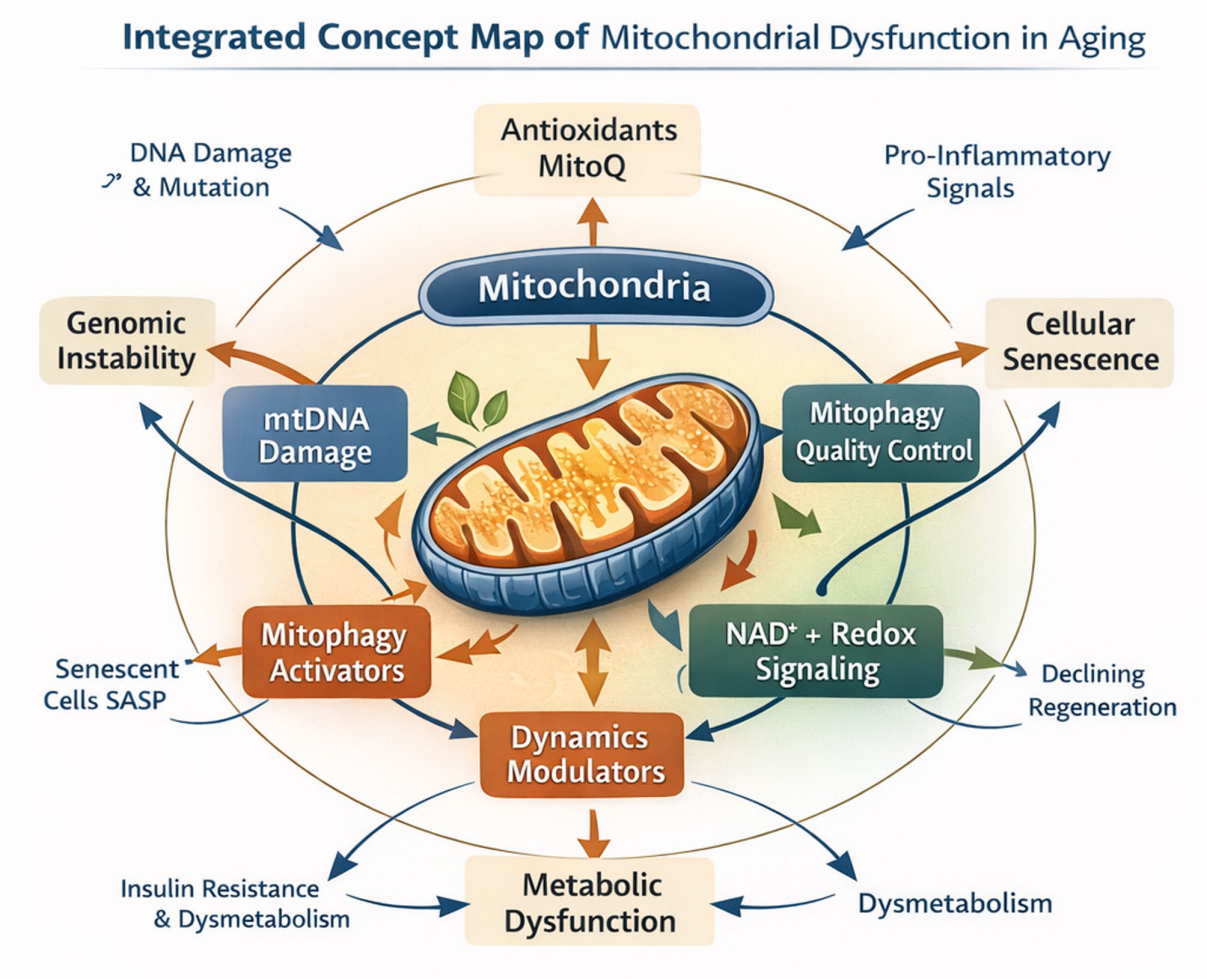
This image contains a combined concept map and a translational map relating to the effects of mitochondrial dysfunction during ageing. These components form a basis for theorizing how mitochondrial dysfunction will influence cellular activity/development as well as ways to treat age-related diseases therapeutically. **The top portion** of the figure represents an **Integrated Concept Map** detailing how mitochondria coordinate interactions with biological pathways associated with age-related fatality markers/traits. The mitochondrial subsystems (mtDNA structural integrity; mitochondrial dynamics, remodeling, and quality control; mitophagy; and NAD+-dependent redox and metabolic signaling) collaborate to form the metabolic base of cellular resilience. Age-related phenotype changes in the cell due to an impairment in communication among mitochondria may have resulted in (1) increased genomic instability; (2) metabolic dysfunction; (3) premature cell senescence; (4) inability to regenerate; and (5) a pro-inflammatory state. The arrows in the figure illustrate how the different types of cellular dysfunction and age-related cellular processes impact mitochondrial failure and, through mitochondrial failure, prompt the development of psychopathology in older persons. Highlighted intervention of interest that includes: (1) mitochondrial targeted antioxidants; (2) stimulation of mitophagy; (3) manipulation of dynamism or dynamics; and (4) targeting NAD + as a pharmacotherapy will facilitate the development of novel therapies to reinstate mitochondrial equilibrium

We suggest that mitochondria should be viewed as an integrated system, with a central hub coordinating the regulation of genomic integrity, metabolic signaling, organelle dynamics, and quality-control pathways. We propose that the decline in the continuity of mitochondrial information flow due to the age-associated changes (to the DNA of mitochondria, the alteration in fission/fusion balance, the impediment in mitophagy, and the decrease in redox signaling associated with nicotinamide adenine dinucleotide [NAD+] levels) contributes to an increasingly serious loss of cellular adaptability and resilience. These failures in manifestations of many hallmark phenotypes of aging (cellular senescence, metabolic dysfunction, impaired regeneration, chronic inflammation, etc.). The systems approach depicted in Fig. 2 illustrates how we can integrate multiple mechanistic aspects of the review. The systems approach highlights the variety of causal interdependencies among the different mechanistic components and identifies several critical points at which therapeutic interventions could be applied.

## Mitochondrial DNA integrity, mutations, and damage

### Mechanisms of mtDNA integrity maintenance

To gain deeper insights into the mechanisms by which cells protect mtDNA, Kang and Hamasaki (2005) conducted an exploratory study [30]. Essential elements for preserving mitochondrial DNA (mtDNA) integrity include the nucleoid structure, various DNA repair mechanisms, the mitochondrial helicase Twinkle, DNA polymerase gamma (POLG), and mitochondrial transcription factor A (TFAM). These procedures help identify, stop, and repair errors such as base misincorporation and damage caused by stress or reactive oxygen species (ROS) during replication. Messenger RNA lacks the mechanisms to repair double-strand breaks, unlike nuclear DNA. For instance, base excision repair is working well, but mismatch repair and nucleotide excision repair are either not working at all or are not working well [31].

### Nature, types, and consequences of mtDNA mutations

mtDNA mutations affect the progression of diseases and the aging process, as Wallace (2010) explained, due to both hereditary and somatic changes. Several types of mutations are recognized, including point mutations, insertions and deletions, large deletions, and rearrangements [3]. Two types of mtDNA mutations are known: homoplasmic and heteroplasmic mutations. Homoplasmic mitochondrial mutations are present in each copy of a cell’s mitochondrial DNA. This suggests that they can be found in both normal and mutant mitochondrial DNA (mtDNA). A higher level of heteroplasmy is associated with deteriorated OXPHOS, raised ROS, and cellular damage. However, in many cases, a low level of heteroplasmy may not be related to impairments in mitochondrial or cellular function. Long-lived postmitotic cells, such as neurons, cardiac muscle, and skeletal muscle, undergo mitosis infrequently, which may make them more prone to mtDNA deletions. Some components of proteins that are coded by mtDNA, like those that make up complex I and complex IV, are necessary for mitochondria to work correctly. Changes to tRNAs or rRNAs that alter the process of making mitochondrial proteins are associated with the decline of mitochondrial function. Wallace emphasized the significance of mtDNA mutations in various mitochondrial disorders and in age-associated decline [3, 32].

### Recent evidence on mtDNA mutations and aging

**Fig. 3:**
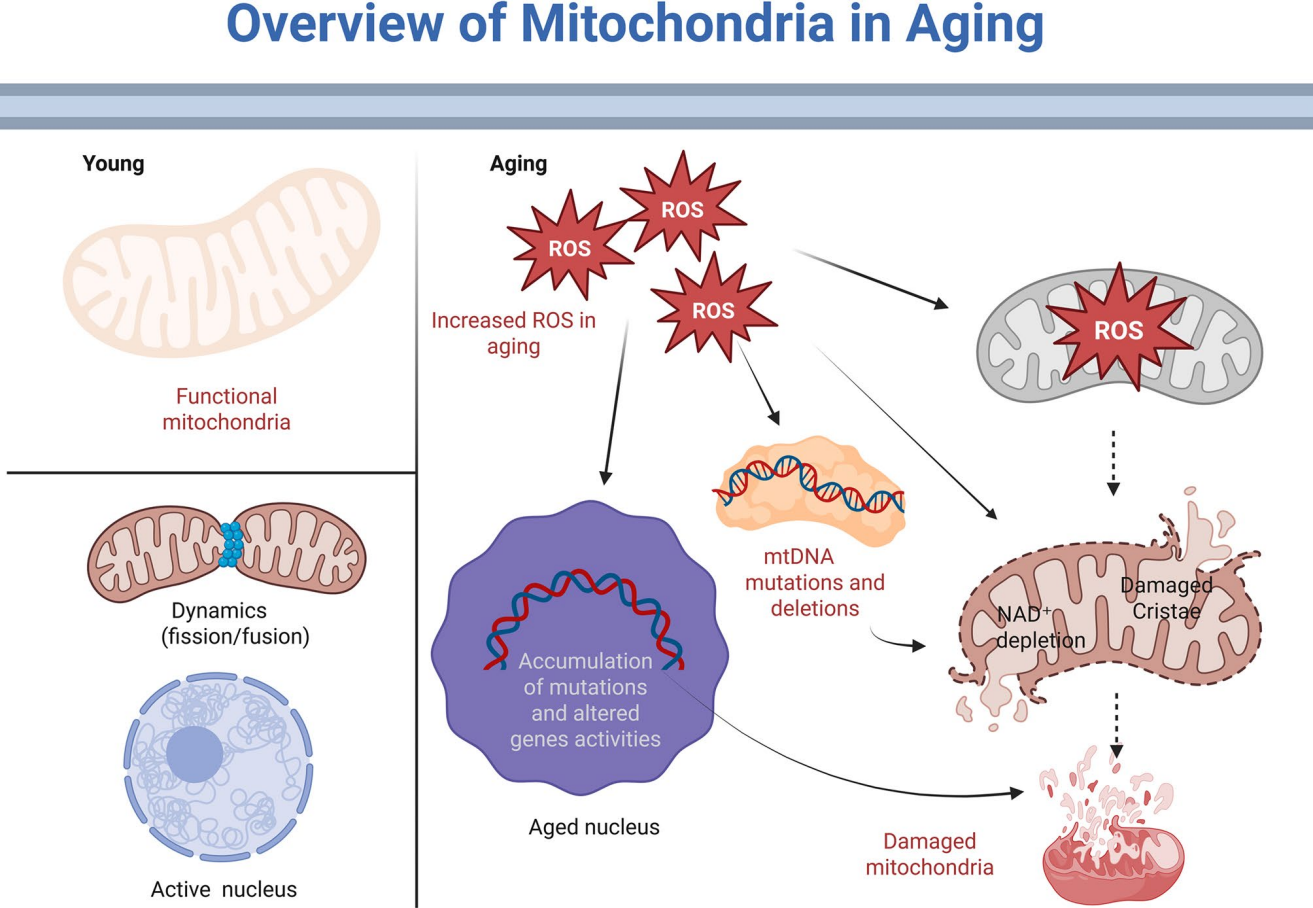
Retaining the integrity of mitochondrial DNA with age. Comparing mitochondria from young and aged cells side by side. In contrast to aging mitochondria, which display mtDNA mutations or deletions, electron transport chain (ETC) malfunction, and elevated reactive oxygen species (ROS) levels, young mitochondria have intact mtDNA, stable oxidative phosphorylation (OXPHOS), and low ROS levels. Only by using deep sequencing can hidden mtDNA mutations with poor heteroplasmy be revealed, as seen in the inset

Some organs, such as the brain, heart, and kidneys, accumulate more mitochondrial mutations than others. These mutations tend to cluster in both coding genes and non-coding genomic regions. Although somatic mutations that occur during a human’s lifetime are not inherited, these mutations have been reported to be associated with suboptimal healing rates in energy-demanding tissues, which are more susceptible to somatic mutations (Fig. 3).

A new study examined mitochondrial DNA variants and their effects on epigenetic and biological aging in young adults. It found that young adults who aged faster, even if they didn’t have any obvious health problems, had higher “functional impact” scores for certain mtDNA variants in coding genes like ND2, ND3, CO3, and CYB [33].

Several factors, including tissue type, cellular turnover, mitochondrial biogenesis, and quality control mechanisms, influence the effects and exact timing of mutations in mtDNA on aging phenotypes. The precise timing of mtDNA mutations’ impact on aging phenotypes depends on tissue type, cellular turnover, quality control systems, and mitochondrial biogenesis. These results elucidate the essential function of the cell in sustaining a functional mtDNA repair mechanism and preserving the mitophage amidst a mutation load [2, 34].

### Integrative mechanisms: damage, repair, mutation accumulation, and threshold effects

The mechanism by which mtDNA can be damaged has been clarified by several studies. It is agreed that exposure to toxins, UV radiation, and metabolic byproducts worsens the oxidative stress induced by ROS produced by OXPHOS. Additionally, ROS stress increases the likelihood of replication errors occurring in mitochondrial DNA polymerase gamma (POLG). Strategies proposed to protect mtDNA must focus on controlling ROS production, ETC leakage. To keep the genome of the mitochondria stable, damaged mitochondria must be removed through mitophagy and other means like base-excision repair, selective destruction of mtDNA and fusing and splitting of mitochondria to reduce mutations [35, 36].

### Therapeutic and research implications

Recent advances in single-cell and ultra-deep sequencing are crucial for the early detection of cryptic or low-level heteroplasmic mutations. This is important because mtDNA integrity plays a vital role in aging, and numerous new treatments and studies are focused on it [37–40]. This could lead to action sooner rather than later. Several studies on “cryptic mutations” have shown that the burden of mutations across tissues may have been underestimated previously [37].

Treatments that aim to improve repair or mitigate damage include enhancing base excision repair, strengthening DNA POLG fidelity, increasing Mitochondrial Transcription Factor A (TFAM) expression, and stimulating mitochondrial biogenesis to reduce the mutation load. Antioxidants and ROS scavengers are additional tools for damage reduction [31, 41].

Scientists are investigating treatments for sporadic disorders through gene editing and mitochondrial replacement. Possible potential applications include reducing the frequency of age-related mutations in mitochondrial DNA. According to evaluations of technologies that alter the mitochondrial base, these methods are becoming increasingly practical [41]. Although conclusive evidence remains absent, recent research has highlighted the importance of regular physical activity, calorie reduction, and the consumption of nutritious meals. These lifestyle modifications enhance the initiation and accumulation of mtDNA mutations by reducing oxidative stress and promoting mitochondrial turnover. Mareckova et al. [33] elucidated that to understand how mtDNA integrity affects aging, it’s crucial to decipher the molecular processes underlying interactions among genetic components, such as mtDNA haplogroups, nuclear gene interactions, baseline mutation load, and the ability to change quality [6, 33, 42].

It is essential to note that both the accumulation of mutations in mitochondrial DNA (mtDNA) and their functional consequences differ across tissue types and tissue compartments. The accumulation of mtDNA mutations and their associated effects occurs at higher rates in post-mitotic tissues than in proliferative tissues. For example, skeletal muscle fibers, neurons, and cardiomyocytes are post-mitotic, long-lived tissues that show gradual clonal expansion of mtDNA deletions and point mutations over time due to low cellular turnover and prolonged exposure to replication-associated errors. In post-mitotic tissues, even small increases in heteroplasmy can adversely affect oxidative phosphorylation and the ability to utilize metabolic substrates, thereby making those tissues more susceptible to aging-related decline [10, 43].

On the other hand, proliferation belongs to tissues that include, among others, the hematopoietic, epithelial, and intestinal compartments. Proliferative tissues exhibit continuous mitochondrial turnover and cell division, as well as the dilution of harmful mtDNA mutations and the introduction of selective pressures that lead to evolution of mtDNA genomic composition over time. The proliferation of progenitor cells gives rise to highly variable patterns of mitochondrial phenotypic expression among proliferative tissues. Within these tissues, high mutation rates (relative to the energy produced) do not necessarily lead to dysfunction. However, mitochondrial reprogramming, reactive inflammatory pathways, and the dynamic nature of stem cells have all been found to impact on how the mitochondrial phenotype is expressed. This impact is heightened in circumstances of chronic stress or when a tissue undergoes further degeneration due to the aging process [2, 44]. Thus, the differences between proliferating and post-mitotic tissues illustrate how mitochondrial dysfunction caused by the aging process depends on a tissue-specific interaction among mtDNA integrity, the tissue’s energy needs, and how well the tissue compensates for decreased energy production.

## Mitochondrial dynamics: fission, fusion, and morphology

Mitochondrial shape and connectivity are governed by an evolutionarily conserved balance between fission and fusion, and this dynamic morphology, in turn, dictates organelle function, quality control, and cell fate. Fusion merges outer and inner mitochondrial membranes to mix matrix contents and maintain bioenergetic competence, a process primarily mediated by the large GTPases mitofusin-1 and − 2 (MFN1/2) at the outer membrane and by OPA1 at the inner membrane [11, 45]. Unlike ‘fusion’, the process of ‘fission’ in mitochondria (in-chain fission) occurs when the protein dynamin-related protein 1 (Drp1) is activated via receptors located on the mitochondrial OM and assembles so that it can then use the energy derived from the hydrolysis of GTP (guanosine triphosphate) to cause constriction/division by wrapping around the two halves of mitochondria after an initial constrictive phase at the points where the two halves come into contact with each other during the process of fusion.

The processes of mitochondrial fusion and fission are interrelated and mutually supportive rather than competing; together, they maximize the cell’s overall effectiveness. The fusion process creates a “pool” for the mitochondria that serves as a buffer against intermittent toxic events or provides energy reserves for energy-depleting situations. The fission process also provides an equal distribution of mitochondria to each daughter cell during cell division and is essential for mitochondrial formation (bioenergetics) and quality control.

As the organism ages, the way mitochondria respond to stress becomes unbalanced, leading to a mixture of healthy and damaged mitochondria. For instance, during acute metabolic stress, the body will attempt to maintain normal mitochondrial function by increasing mitochondrial hyperfusion or even further development of the abnormal mitochondrial structure [46]. One of the primary roles of mitochondrial fission is to separate damaged mitochondria from healthy mitochondria. Localized fission creates a new, physically separated, and depolarized daughter mitochondrion, which can then be selectively removed from the healthy daughter mitochondria by receptor-mediated or PINK1-Parkin-dependent mitophagy.

As the organism ages, the separation between fission and mitophagy is altered, causing an increase in dysfunctional mitochondria due to the presence of abnormal fission and little or no fission to provide for the removal of damaged mitochondria through mitophagic processes [47, 48]. These findings support the notion that the regulation of mitochondrial dynamics is based on the surrounding environment and that disease results from the breakdown of coordinated regulation regarding the integration of mitochondrial fission, quality control, and mitochondrial fusion.

Fusion and fission work together to regulate both mitochondrial morphology and size. They also affect mitochondrial bioenergetics and the production of reactive oxygen species (ROS). Ultimately, fusion and fission dictate how a mitochondrion will respond to mitophagy and/or apoptosis.

Regulation is a complex process at the molecular level. A variety of post-translational modifications (PTMs), proteolytic processing, alternative splicing, and MFNs regulate the GTPase activity of OPA1 and Drp1. For instance, OMA1- and YME1L-dependent proteolysis produces both long and short forms of OPA1. The ratio of these isoforms determines how well inner membranes fuse and how stable the cristae are [49, 50]. Phosphorylation (Ser616 activation, Ser637 inhibitory in many contexts), SUMOylation, ubiquitination (Parkin/PINK1 pathway), and S-nitrosylation control Drp1’s activity. The ability of Drp1 to import and disassemble mitochondria is affected by these changes. Recent studies have connected the acetylation of OPA1 and related regulators to aging-related organ dysfunction and loss of cristae integrity [51]. The fusion of fragments of mitochondria through mitophagy processes from the PINK/PK gene pathway ensures that all damaged, broken or otherwise defective parts (i.e. mitochondria) will be properly processed via this pathway as well as through other mitochondrial quality control mechanisms [52, 53].

In terms of bioenergetic and signaling outputs, morphology is highly related. Faster oxidative phosphorylation, stable membrane potential, and the ability for organelles to share mtDNA and proteins are all benefits of fusion. Fragmentation, in contrast, reduces respiration efficiency, increases ROS levels, and prepares mitochondria for autophagic clearance or death. New cellular-level, high-resolution research shows that equilibrium changes with age, leading to increased fragmentation and compromised cristae integrity across different tissues. Stress tolerance is reduced, and this mechanism impairs ATP generation. Furthermore, stem cell division results in an unequal distribution of healthy and damaged mitochondria to daughter cells, impairing their ability to divide and maintain tissue homeostasis [54]. This process connects mitochondrial dynamics to cellular aging.

Their association with human disease phenotypes highlights the translational relevance of genetic changes that influence dynamics. Mutations in MFN2 cause axonal degeneration in Charcot-Marie-Tooth disease type 2 A, while mutations in OPA1 cause autosomal dominant optic atrophy with cristae abnormalities and retinal ganglion cell loss [49, 55]. In models of neurodegeneration, cardiac ischemia-reperfusion injury, metabolic disease, and acute organ injury, altered Drp1 signaling and excessive fission are seen, making Drp1 and its receptors potential therapeutic targets [50, 52]. It is worth noting that the same proteins behave differently across tissues and disease states, limiting therapeutic windows.24 Inhibiting fission or fusion completely can be harmful because of their essential roles in development and basal homeostasis [52].

**Fig. 4:**
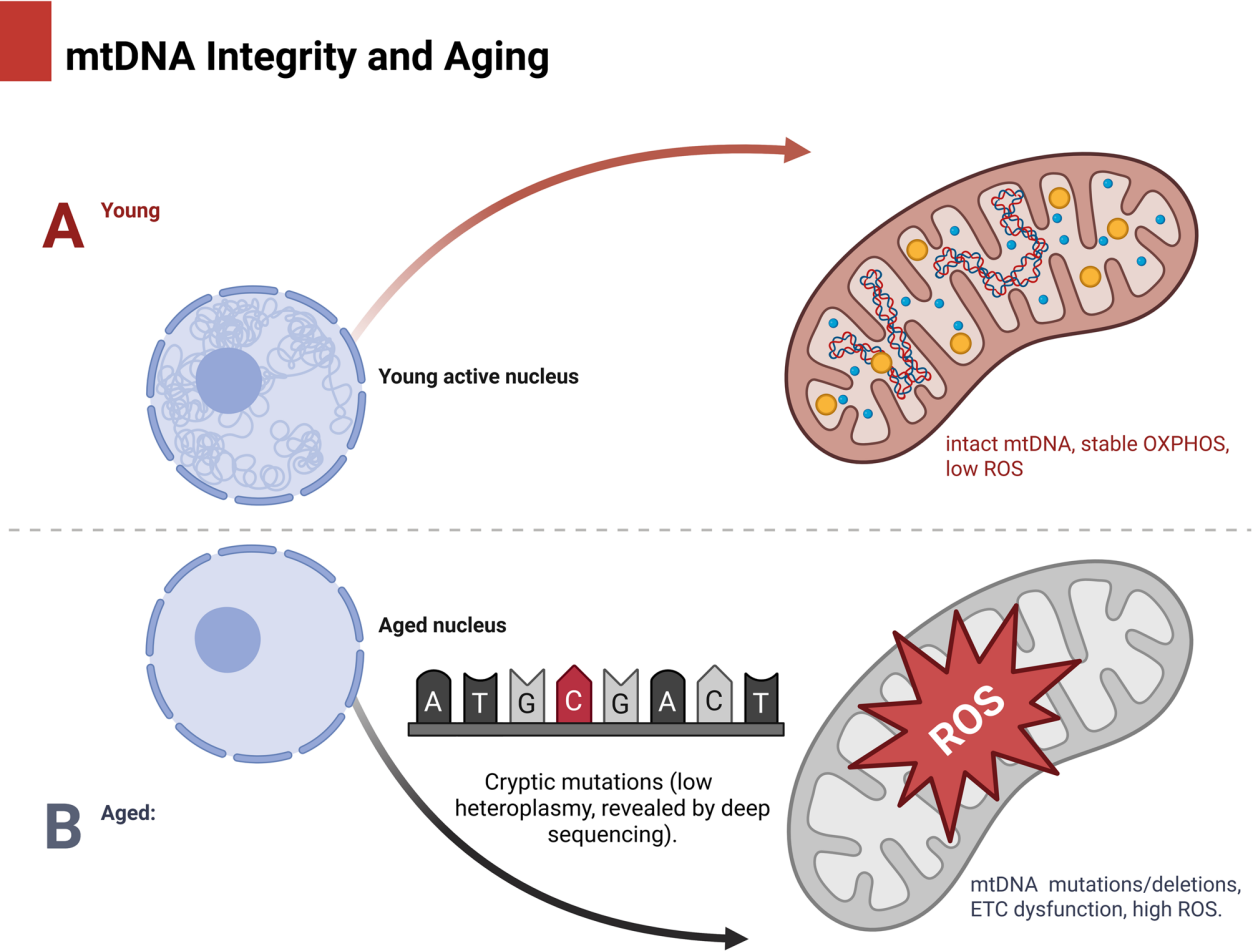
Aspects of mitochondrial dynamics include morphogenesis, fission, and fusion. Fission and fusion cycles are depicted graphically in the mitochondrial network. (**A**) During fission, DRP1 is transported from the cytosol to the OMM by means of MFF, FIS1, and MiD49/51. It is DRP1 that regulates this procedure. Then, to aid with constriction, it forms a ring shape. (**B**) Fusion: mitofusins (MFN1/2) use trans contacts to facilitate OMM fusion, and OPA1 and cardiolipin drive IMM fusion. When everything is in harmony, adaptive morphology can take place; when things are out of whack, it’s associated with aging. Annotated lists of human diseases related to MFN2 and OPA1 mutations are available in the appendices

Numerous approaches have been developed to transform basic concepts into practical methods for medical practice. Small molecules and peptides that modulate fission/fusion have been employed in preclinical models: Mdivi-1 (initially identified as a Drp1 inhibitor) has produced inconsistent outcomes and off-target effects, while the peptide P110 — engineered to obstruct the Drp1–Fis1 interaction — has demonstrated an organ-protective effect in models of ischemia-reperfusion and acute kidney injury by mitigating maladaptive mitochondrial fragmentation [53, 56]. Testing preclinical models is currently underway for gene therapy options, including AAV-mediated delivery of the MFN1 & MFN2 gene, OPA1 gene, as well as RNA silencing and CRISPR genome editing. The primary focus of all these approaches will be to prevent any potential adverse impact on mitochondrial dynamics, as most tissues have distinct requirements for these organelles based on their overall function (a muscle cell has different needs than a neuron) [52, 53]. Finally, stratifying patients by genetic background (MFN2/OPA1 variants, mitophagy-related mutations) and by tissue-specific vulnerability may enable personalized interventions that balance benefits and risks (Fig. 4). Recent progress in small-molecule screening and structure-guided design of modulators has facilitated isoform- or site-specific interventions aimed at fine-tuning activity rather than completely inhibiting essential GTPases [33, 51].

Additional targets for translational research include molecular mechanisms that occur before dynamics. Modulators of OPA1 processing (OMA1/YME1L), post-translational modifying enzymes (kinases such as CDK1 and CaMK, and phosphatases like calcineurin), and ubiquitin ligases (Parkin) can indirectly rebalance morphology and quality control [11, 52]. Metabolic and lifestyle interventions — exercise, caloric restriction, and NAD+-boosting strategies — influence acetylation, sirtuin activity, and mitochondrial biogenesis and have been shown to favor more fused, functionally resilient mitochondria in preclinical and some human studies [52, 57]. Such systemic interventions are attractive clinically because they act broadly on bioenergetic networks and mitohormetic signaling, but their specificity for correcting pathological dynamics is limited, and patient responses are heterogeneous.

Safety and delivery issues are additional challenges in clinical development. It is better to partially and temporarily control fission and fusion rather than completely stop them because they are so important. It is uncertain whether prolonged systemic inhibition of Drp1 is more advantageous than short-term interventions, such as ischemia-reperfusion, following acute injury. Tissue-targeted dispersion methods, such as peptide delivery systems or organ-selective AAVs, and inducible expression can reduce the likelihood of off-target effects. Biomarkers that indicate mitochondrial shape or turnover in living organisms will make it much easier to select the right patients and determine the correct dose. Functional metabolic signals, imaging tests of the potential of organelle membranes, or circulating cell-free mitochondrial DNA could be some of these biomarkers [52, 53]. By categorizing patients based on their tissue-specific susceptibility and genetic predispositions (such as MFN2/OPA1 variants and mitophagy-related mutations), it may be feasible to administer personalized treatments when the benefits outweigh the risks.

In summary, several approaches can treat this condition by targeting the molecular machinery that controls mitochondrial division, fusion, and shape changes. Drp1 and its receptors, MFN1/2 and OPA1, their proteases, and PTM enzymes, as well as upstream signaling networks, are all part of this machinery. Recent studies indicate that age-associated alterations in mitochondria encompass the loss of cristae and fragmentation, asymmetric segregation, and the modulation of effective dynamics in disease models, thereby offering substantial support for translational research [54, 56, 58]. However, instead of a blanket ban, responsible clinical translation requires context-sensitive modulation, careful patient selection based on biomarkers, and strategies that combine agents targeting morphology with therapies that enhance metabolic function and mitophagy to restore organelle integrity while preserving mitochondrial plasticity [52, 53].

## Mitochondrial function, bioenergetics, and regulation

Bioenergetics happens mostly in mitochondria. That’s where the processes that control cellular function and fate come together: cellular metabolism, ATP production, redox balance, and signaling pathways. Three different regulatory mechanisms control how mitochondria are used for their metabolism through biogenetic pathways, creating energy and grading the functional capacity of the mitochondria. Understanding these regulatory networks is crucial for advancing therapeutic strategies in aging and disease, as well as for fundamental biological research [59, 60].

**Fig. 5:**
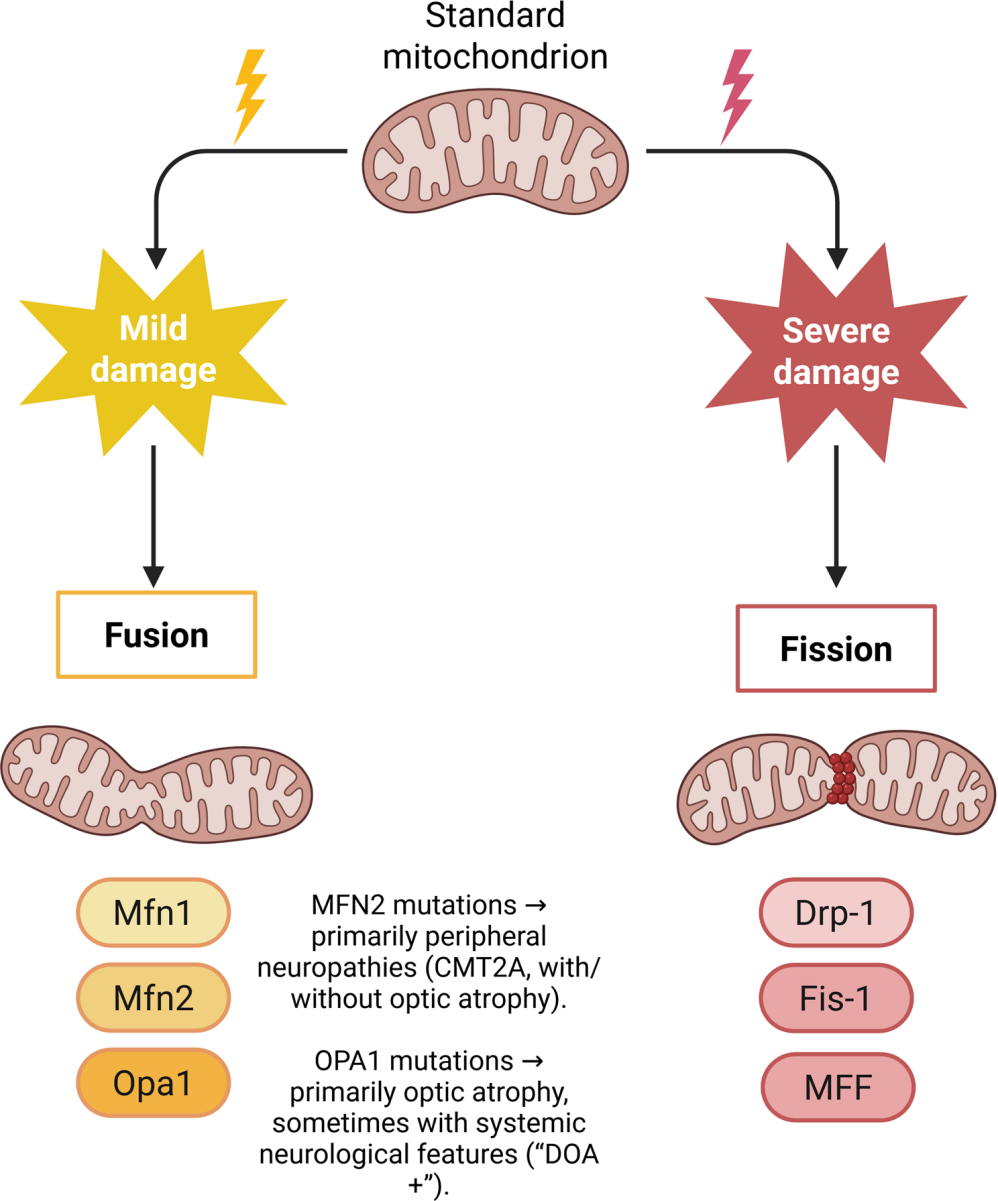
In this image, we can see the mitochondrial electron transport chain in action. Complexes I–IV generate energy by pumping protons across the membrane. These complexes are pathways for electrons to migrate from metabolic substrates. Complex V (ATP synthase) is powered by a proton gradient generated in this way

The OXPHOS system, which is made up of the embedded ETC complexes I–V in the inner mitochondrial membrane, is the most essential part of mitochondrial bioenergetics. When NADH and FADH2 give up electrons (Fig. 5), protons move across the membrane and form molecular oxygen. ATP synthase creates an electrochemical gradient in complex V. The production of energy by mitochondria requires the cooperation of the products derived from both the mitochondria and the nucleus. Therefore, when the mitochondria and nuclear genes fail to work together, it usually correlates with some kind of metabolic or degenerative disease [61]. When any of these components fail, ATP synthesis is hindered, ROS levels rise, and cell viability diminishes [57].

Mitochondrial biogenesis is regulated by a synchronized communication process between the nucleus and the mitochondria. Peroxisome proliferator–activated receptor gamma coactivator 1-alpha (PGC-1α) plays a crucial role in regulating this process by activating mitochondrial transcription factor A (TFAM) when interacting with nuclear respiratory factor 1 (NRF1) and nuclear respiratory factor 2 (NRF2). Energy-sensing pathways also influence PGC-1α’s activity, thereby accelerating the replication and transcription of mtDNA [61]. The AMP-activated protein kinase (AMPK) is one example. It turns on when energy levels drop. The SIRT1 deacetylase, which requires NAD+, enhances PGC-1α function [62]. The metabolic flexibility and energy supply of the cell, as well as the mitochondrial redox homeostasis will be regulated by these pathways together as well [52].

The functions of mitochondria are maintained by several factors, of which SIRT1 (a sirtuin that alters acyl-lysine modifications) is one. It primarily regulates the activity of enzymes involved in the TCA cycle, fatty acid breakdown, and ROS removal, thereby protecting against oxidative damage. For example, altering the breakdown of succinate dehydrogenase and lysine by SIRT3 and removing acetyl groups from manganese superoxide dismutase (MnSOD) are crucial for activating it and thus protecting cells from oxidative damage. These effects demonstrate that sirtuins act as metabolic rheostats, modulating energy expenditure and mitochondrial function [62].

Researchers have determined how the mechanistic target of rapamycin (mTOR) regulates protein synthesis, cell death, and mitochondrial function in response to signals from growth hormones and nutrients. The primary function of mTOR complex 1 (mTORC1) is to stimulate mitochondrial biogenesis by facilitating the translation of PGC-1α and activating transcriptional pathways that enhance oxidative metabolism. Also, blocking mTORC1 with Rapamycin or not getting enough nutrition can speed up autophagy and mitophagy, two processes that help remove damaged mitochondria. Maintaining a balance between anabolism and catabolism is crucial for controlling aging and age-related diseases. Controlling mTOR improves health span in many species; however, an overactive mTOR accelerates deterioration [63, 64].

**Fig. 6:**
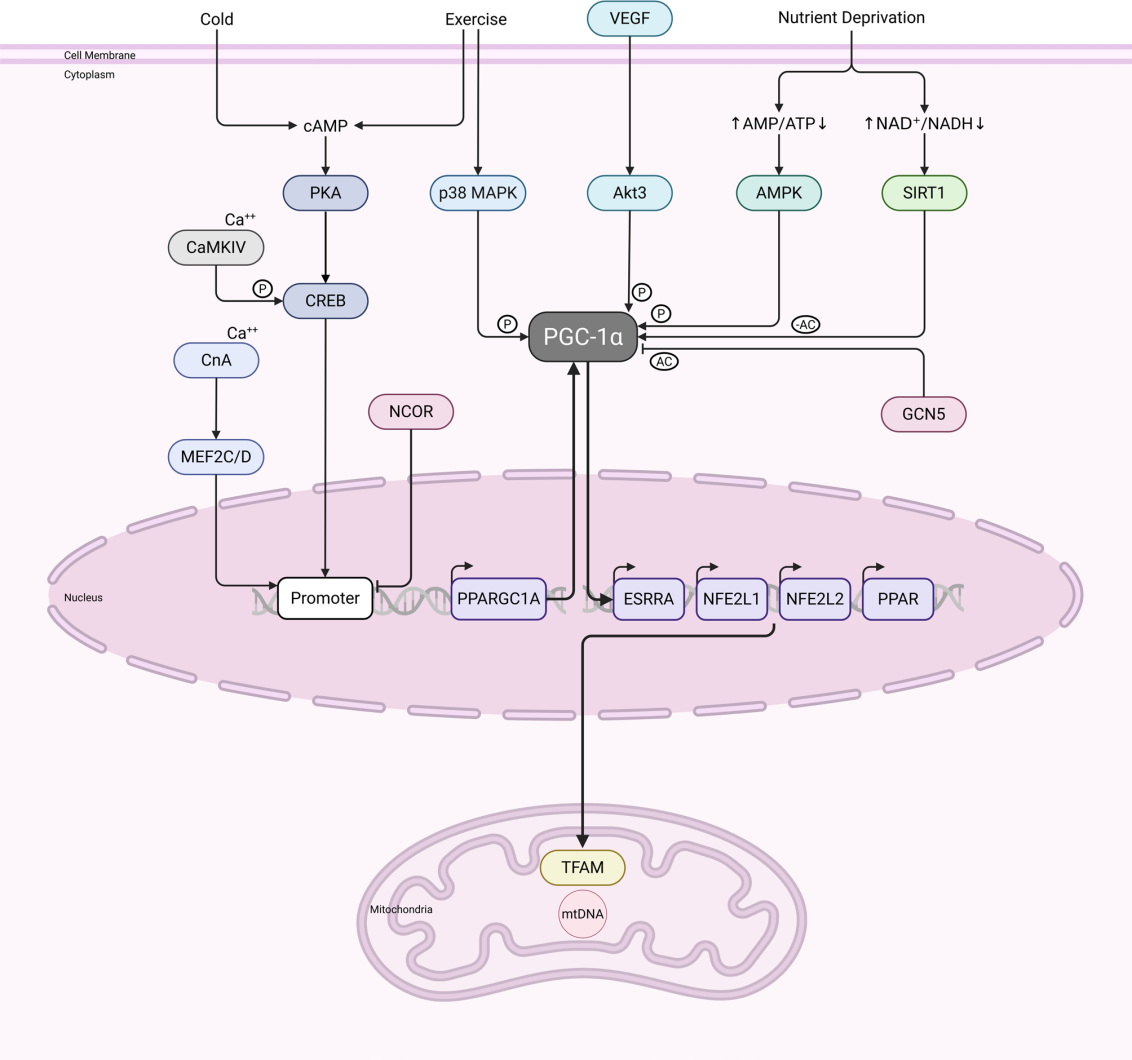
Managing bioenergetics and mitochondrial function. A schematic depicting the axis of PGC-1α-NRF1/2-TFAM. Nuclear transcription and TFAM-mediated mtDNA replication are controlled by AMPK, SIRT1, and mTORC1, which are energy and nutrient sensors that collaborate through PGC-1α. Modifications to this axis can result from dietary changes, increased physical activity, or medications that increase NAD+ levels or activate sirtuins, or from rapamycin. Stronger antioxidant defenses increased mitochondrial biogenesis, and improved OXPHOS are the outcomes

The elucidation of mitochondrial regulatory networks has facilitated the discovery of novel therapeutic pathways. Scientists are investigating sirtuin activators (such as resveratrol and NAD+ precursors), AMPK agonists (such as metformin), and mTOR inhibitors to determine whether they can enhance mitochondrial function and slow the aging process. In metabolic and neurodegenerative disorders, strategies that enhance PGC-1α signaling or promote mitophagy could help maintain mitochondrial integrity [64–66]. However, this is promising because mitochondrial responses vary across different tissues, and excessive stimulation of biogenesis or mitophagy could have adverse effects [67]. To bridge the gap from the lab to the bedside, we need to enhance precise delivery, biomarker development, and patient stratification, as illustrated in Fig. 6.

## Mitophagy, quality control, and turnover

Mitophagy is an essential quality-control mechanism of the mitochondria that selectively removes dysfunctional or unneeded mitochondria to maintain healthy cells and ultimately achieve a stable state of proliferation [13, 49]. The major regulatory pathway for mitophagy is that of the PINK1-Parkin pathway. This is initiated when mitochondrial membrane potential is compromised, leading to PINK1 stabilization on the outer mitochondrial membrane, followed by Parkin recruitment and the consequent widespread ubiquitination of outer membrane proteins. These events serve to designate dysfunctional mitochondria for clearance via the autophagy process [68–70]. In conjunction, mitophagy can also occur via receptor-mediated mechanisms in response to hypoxia or during development through the activity of proteins such as BNIP3, NIX/BNIP3L, and FUNDC1, which directly associate with LC3 [71–73]. The initiation and subsequent flux of mitophagy is subject to multiple sources of regulation based on nutrient and energy availability: starvation state induces activation of AMPK as a promoter of autophagy, whereas mTORC1 serves as a principal inhibitor of autophagy in a nutrient-rich state [74–76]. Additionally, the execution of mitophagy requires that damaged mitochondria be degraded by competent lysosomes, as well as coordination via transcription factors such as TFEB, which initiate autophagosome-lysosome fusion and regulate lysosomal capacity for degradation [77, 78]. As a person ages their body’s ability to remove old, damaged mitochondria declines; this results in an overwhelming amount of defective mitochondria in the organism, an increase in oxidative stress within the cells and metabolism becomes more constrained due to the increase amounts of defective mitochondria in the organism [2, 79].

**Fig. 7:**
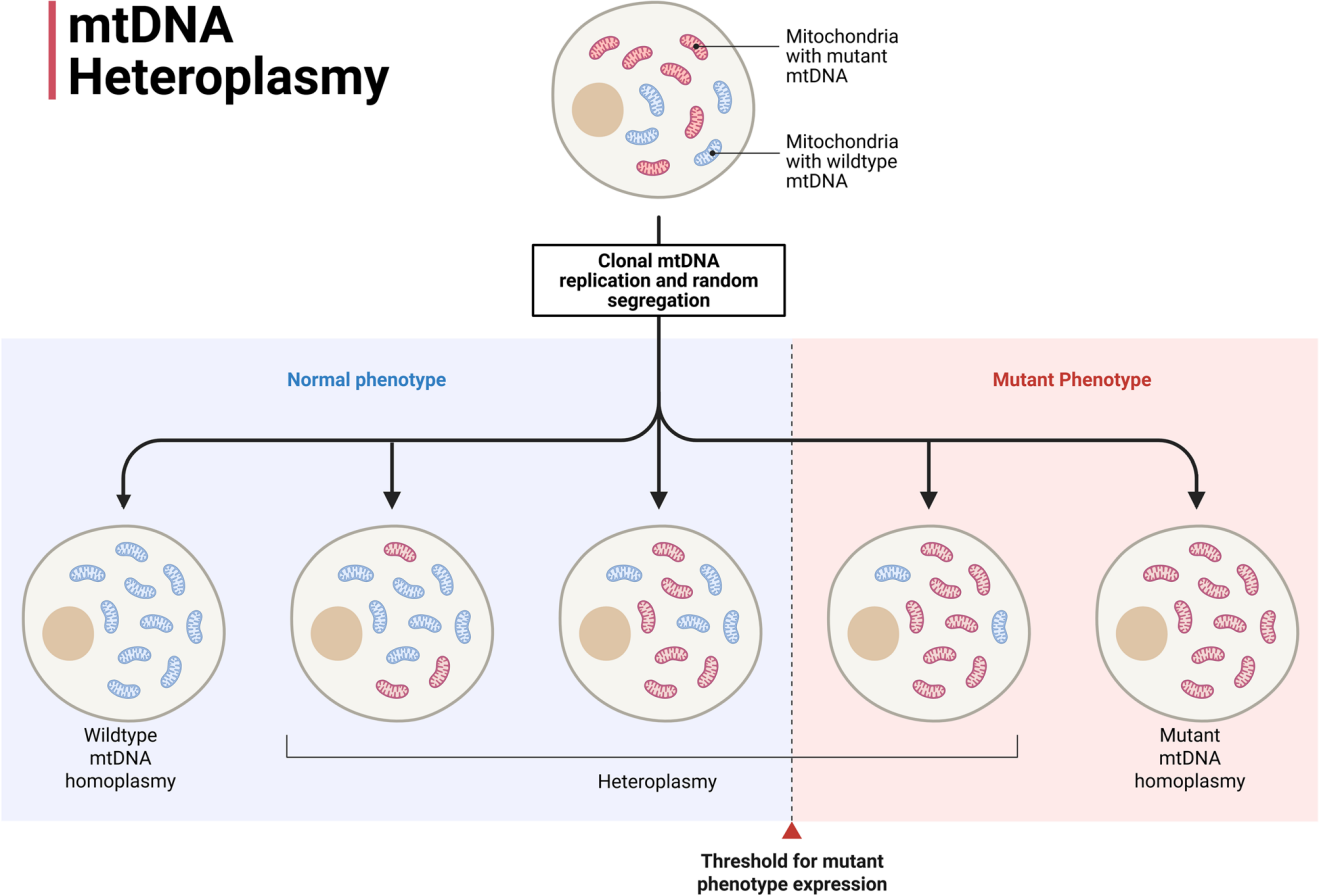
Multiple copy-number anomalies in mitochondrial DNA. The presence of both wild-type and mutant mitochondrial DNA molecules within a single cell or mitochondrion results in heteroplasmy. Shows how heteroplasmy levels change over time and how they affect OXPHOS capacity, with a focus on threshold effects in disease and aging

Additional methodologies, including live-cell imaging of LC3 and autophagosomes, profiling the ubiquitin/protein ubiquitome, high-depth sequencing of mtDNA heteroplasmy (Fig. 7), and assays for MDV formation, elucidate the differentiation between selective and wholesale removal, tissue specificity, and age-related variations in turnover.49,50 These studies show that mitophagy rates decrease with age across many tissues. They have also demonstrated that compensatory pathways, such as MDVs, may be effective when bulk mitophagy is not [80, 81].

Combining mitophagy enhancers with drugs that boost mitochondrial biogenesis, such as PGC-1α activators, exercise, and NAD+ elevation, can replace damaged mitochondria with healthy ones. This makes combination approaches more attractive than single-agent strategies. At the same time, drugs that help proteostasis or lower inflammation help protect tissues. Lysosomal enhancers are proposed as an alternative to Parkin agonists for a new therapeutic strategy in Parkinson’s patients with autophagy gene mutations, lysosomal acidification, or genetic abnormalities. Currently, mitophagy biomarkers are the focus of many recent studies. Using these markers, the process of detecting significant defects in mitochondrial function, such as mt-Keima-like signals, mitochondrial DNA patterns in moving cells, mitophagy protein fragments, or metabolomic signatures, is demonstrated. Moreover, these markers help adjust therapeutic doses and monitor progress in clinical trials [81–86].

## NAD⁺ metabolism and mitochondrial regulation

Nicotinamide adenine dinucleotide (NAD-) is essential for cellular metabolism and signaling. This redox cofactor is needed by dehydrogenases in glycolysis, β-oxidation, and the tricarboxylic acid (TCA) cycle. It also acts as a limiting co-substratum for enzymes such as ADP-ribosyl cyclases (e.g., CD38), PARPs, and sirtuin deacylases. NAD + is involved in both cellular and mitochondrial processes, so its levels affect mitochondrial bioenergetics, quality control, stress responses, and communication between the nucleus and mitochondria. Multiple metabolic pathways will be affected by a reduction in the availability of NAD + as we age or develop chronic health conditions. For example, sirtuin activity goes down (which affects mitochondrial protein acetylation and function), PARP activity goes down (which slows down DNA repair), and Ca²+/cADPR signaling changes (through CD38). All of these changes could cause the mitochondria to stop functioning correctly. Researchers have demonstrated in preclinical aging models that nicotinamide mononucleotide (NMN) and other NAD+ precursors can enhance various age-related characteristics in mice. This means that altering how the body uses NAD + can affect how organisms age and how well their mitochondria function [87].

Mitochondrial function and NAD metabolism are interdependent, with bidirectional redox regulation and signaling. Age-related declines in NAD availability impair NAD-dependent enzyme (sirtuin) activity, leading to disrupted mitochondrial protein acetylation, reduced oxidative phosphorylation efficiency, and decreased ability to respond to stress and to functionally remove dysfunctional mitochondria (mitophagy). Disruption of mitochondrial respiration due to impaired electron transport chain function, altered substrate utilization, and impaired redox shuttle activity disrupts the cellular NAD/NADH ratio and depletes NAD at the same time, leading to a decreased ability to regenerate it [14, 88]. Therefore, impaired mitochondrial respiration also contributes to the decline in NAD, creating a positive feedback loop that reduces the mitochondria’s ability to adapt over time.

The degree to which this positive feedback loop affects mitochondrial function varies by tissue. Factors such as mitochondrial density, metabolic requirements, capacity for NAD biosynthesis, and expression of the main NAD-consuming enzymes (CD38, PARPs) determine how different tissues experience this phenomenon. For instance, tissues with a high metabolic requirement such as skeletal muscle, brain, and heart, tend to have a greater effect from NAD/mitochondrial homeostasis disruption than do other tissues, whereas tissues that are highly regenerative may have an advantage by stimulating their salvage pathways [89, 90]. Differences in tissue characteristics further clarify why responses to NAD-boosting interventions vary among tissues, underscoring the importance of interpreting these results in the context of mitochondrial environment and inflammatory load, as well as baseline NAD turnover for each tissue.

A series of steps, such as making, storing, using, breaking down, and moving NAD+ throughout the body, help maintain its levels. Interventions focus on the salvage route, which is the most critical pathway in many mammals [91]. Exogenous precursors, such as nicotinamide riboside (NR), nicotinic acid (NAc), and non-myosin monophosphate (NMN), enter these pathways at different points and have different pharmacokinetic properties. Cells need NAD + for a lot of things, like turning on Sirtuins (SIRT1 in the nucleus/cytosol and SIRT3 in the mitochondria, to name a few), turning on PARPs (which are turned on when DNA is damaged), and breaking down NAD + by the membrane enzyme CD38. If any of these processes are very active, they can deplete cellular NAD+. Always remember that NAD+ pools aren’t entirely separate. There are three different pools of NMNAT, and each one is controlled by a different way of moving or taking it in: one in the nucleus, one in the cytosol, and one in the mitochondria. Consequently, deficiencies related to mitochondrial function may remain undetected in global evaluations of NAD⁺ [92].

Sirtuins can control whether NAD+ levels go up or down. SIRT1 activates PGC-1α, the main coactivator of mitochondrial biogenesis, by deacetylating it. As a result, TFAM encourages the transcription and replication of mitochondrial DNA and the transcription of nuclear-encoded mitochondrial genes. Hypoxia decreases sirtuin activity, leading to hyperacetylation of mitochondrial proteins, compromised respiration, increased ROS levels, and diminished mitophagy or biogenesis responses [91]. Mitochondrial SIRT3 deacetylates and activates enzymes that play roles in the TCA cycle, fatty acid oxidation, and antioxidant defenses. Conversely, increasing NAD⁺ levels via precursors or reducing the levels of key NAD⁺ consumers can enhance sirtuin activity, mitochondrial enzyme function, and mitochondrial resilience [87, 93].

The results of translational research have been inconsistent, yet beneficial, as they have rapidly progressed from animal studies to human trials. Research involving humans has demonstrated that oral administration of NR or NMN can elevate NAD⁺ metabolites in blood and tissues and induce favorable alterations in genes and biomarkers in blood and skeletal muscle [94–97]. Nonetheless, the impact on clinical outcomes, such as strength, metabolic health, and cognition, has shown variability across populations and research methodologies.

To sustain elevated NAD+ levels and enhance the effectiveness of precursor supplementation, it is essential to inhibit significant NAD+ consumers, including CD38 (which escalates with age and inflammation) and hyperactive PARP (which arises from chronic DNA damage). To enhance NAD⁺ kinetics and tissue specificity, preclinical and early clinical trials are currently underway for pharmacological CD38 inhibitors, PARP modulators, and small molecules that either increase NAMPT expression or stabilize NMNAT activity. There is also considerable interest in multi-ingredient nutraceutical formulations that aim to make the salvage route easier to access and utilize in various locations. There are also “systems” approaches that mix precursors with cofactors such as resveratrol, pterostilbene, and polyphenols [15, 90].

When you look at both functional readouts (like mitochondrial respiration, the acetylation status of sirtuin targets, and mitophagy markers) and the levels of NAD+ precursors and intermediates in plasma and target tissues (like skeletal muscle biopsies), you get a better picture. There are only a few clinical endpoints; however, trials that utilize mechanistic endpoints, such as NAD+ metabolomics, sirtuin target deacetylation, and mitochondrial respiratory capacity, have made it easier to demonstrate that the target is being engaged [98].

Comprehensive, long-term randomized trials are essential for ascertaining efficacy in aging-related outcomes and for detecting rare or delayed adverse effects. Nonetheless, short-term trials typically demonstrate favorable tolerability for NR and NMN. Other unknowns include the safety of long-term use, potential off-target effects (such as changes in methylation pathways driven by higher NAM turnover), and varying effects across age groups and disease states. It is imperative to exercise caution when administering NAD+-boosting therapies to patients with cancer, inflammatory disorders, or any condition in which altering NAD+ metabolism may lead to unforeseen consequences, given NAD+’s essential role in regulating DNA repair and immune signaling [94, 95].

Clinical translation will likely require targeted approaches rather than a one-size-fits-all approach. To determine which groups of patients are most likely to benefit, you can categorize them by initial NAD+ levels, inflammatory load (which triggers CD38), PARP activation, mitochondrial dysfunction phenotype, and genetic makeup (including variations that alter NAD+ salvage enzymes). The most effective method to elevate NAD+ levels may be to combine it with dietary and exercise modifications known to enhance mitochondrial biogenesis and sirtuin pathways. They increase mitochondrial bulk and function by providing additional substrates and transcriptional programs. This, in turn, elevates NAD+. Ultimately, customized delivery platforms can enhance the effectiveness of therapies while reducing the likelihood of side effects and systemic exposure. These platforms comprise tissue-selective prodrugs, targeted nanoparticles, and gene therapy methodologies that enhance NMNAT expression in specific tissues [98].

NAD⁺ metabolism is an essential part of controlling how mitochondria work. It is both a redox cofactor and a necessary co-substrate for sirtuins and other signaling enzymes. Preclinical studies indicate that replenishing NAD⁺ can improve mitochondrial bioenergetics, proteostasis, and stress resistance. Initial human trials suggest that these pathways can be pharmacologically altered. To safely and effectively translate NAD⁺ biology into clinical interventions, it is necessary to accurately determine dosages, carefully select patients, develop mechanistic biomarkers to identify targets, prioritize long-term safety, and likely employ combinatorial strategies to improve mitochondrial biogenesis and quality control [87, 91–93].

## Therapeutic interventions and translational approaches

**Fig. 8:**
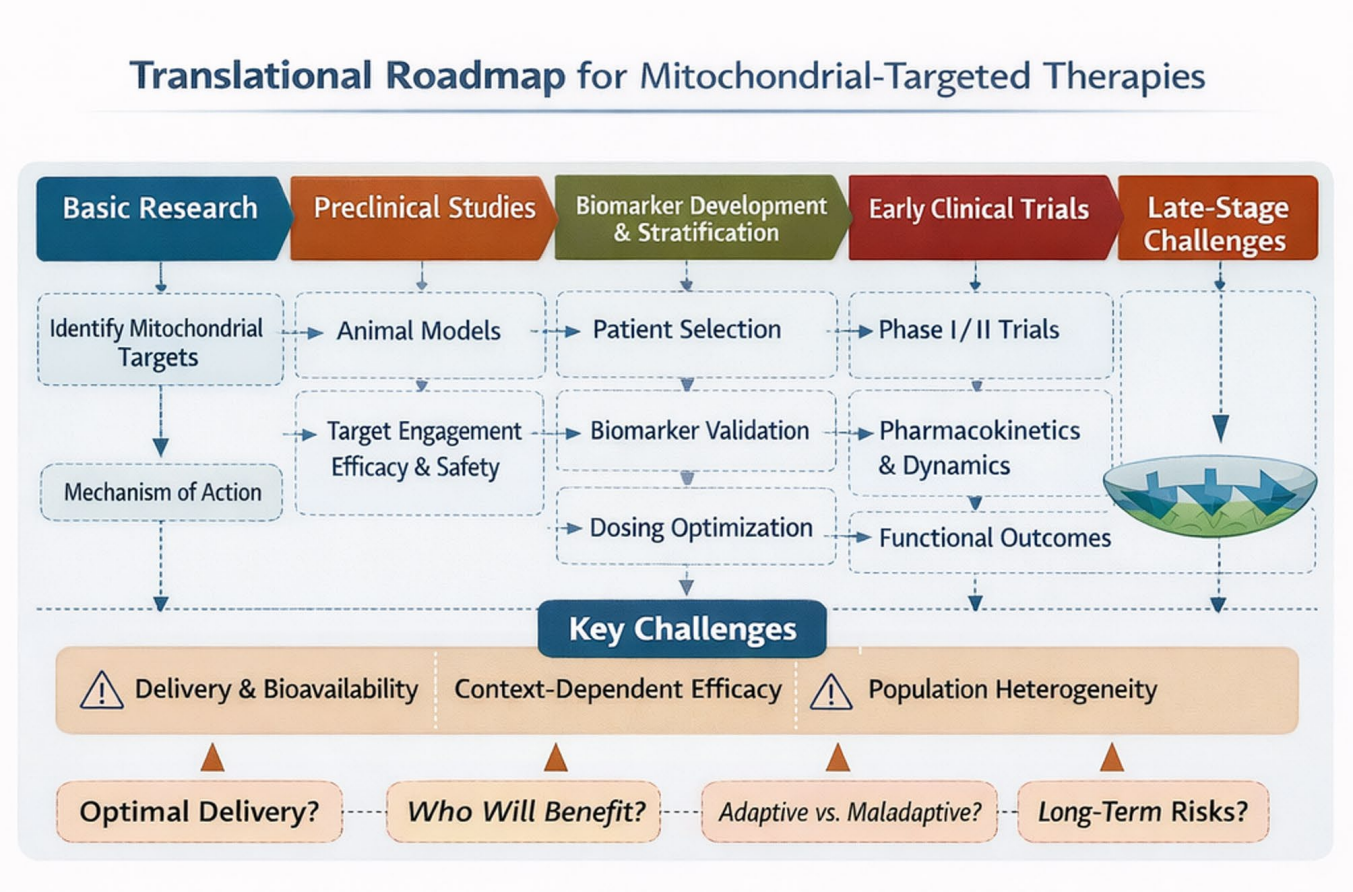
The translational roadmap for mitochondrial-targeted biomarker/medication is illustrated in the **lower half of the image**. The table provides a framework for reviewing the steps taken, from basic (e.g., target identification and mechanistic elucidation) to clinical intervention (e.g., validation of biomarker development and determination of patient cohorts for studies), across various stages of drug development. Roadmap milestones for fibrotic or mitochondrial-targeted biomarker/therapeutics include (beginning of clinical development) validating target engagement, determining optimal dosages, and assessing functional outcomes prior to clinical intervention. Three nodes illustrate the most common obstacles to producing successful clinical outcomes; problems related to biodistribution (administering drugs to patients), administering the wrong drugs based on individual efficacy (determining the context in which to give drug), the use of heterogeneous patient populations, and issues related to the long-term safety of innovative therapies. The translational approach based upon mechanistic research related to the role of mitochondria in the ageing process and cellular degeneration provides a systems-level framework for developing targeted therapeutics to combat mitochondrial dysfunction that results in ageing

The need for a systematic roadmap to navigate from the discovery of mitochondrial function and its mechanisms to their respective clinical applications has led us to develop a structured continuum concept. It is critical that we have a roadmap for translating research into clinical trials so that we understand all required steps along the way, and ultimately, we can complete the translation to reach a fast ability to identify and evaluate the efficacy of potential clinical translation. The validation points along this continuum include evidence of successful mitochondrial engagement, restoration of adaptive responses, and the ability to measure improvement in clinical outcomes. At the same time, many challenges remain to be overcome to execute this continuum. These challenges include difficulties in delivering therapies targeting mitochondria, differences in efficacy across tissue types and other contextual factors, the wide variety of patients with mitochondrial disease or dysfunction, and long-term safety concerns. The image demonstrates how each phase of the translational continuum can cause potential therapies to succeed or fail within the context of their respective clinical studies, based specifically on whether essential validation stages and corresponding contextual challenges were considered or not (Fig. 8).

Mitochondrial dysfunction is central to aging, senescence, and various chronic diseases, prompting the development of therapeutics targeting mitochondria to progress from a mechanistic understanding to initial clinical application. Increased production of ROS, as a result of damage to the mitochondrial electron transport chain (ETC), leads to cellular senescence, the release of damage-associated molecular patterns (DAMPs), and the initiation of a cycle of oxidative stress and inflammatory response that contributes to further injury within affected tissue. The three main goals of mitochondrial health promotion therapy are as follows: (1) lowering the risk of mitochondrial damage; (2) improving clearance of damaged mitochondria through mitophagy; and (3) restoring mitochondrial function through biogenesis, metabolic support, or direct organelle-targeted interventions [87, 99].

### Mitochondrial dysfunction-associated senescence (MiDAS): distinguishing mitochondrial-specific senescence from canonical DNA damage-induced senescence

Initially viewed as a long-lasting precluding mechanism triggered by constant nuclear DNA damage and activation of the DNA Damage Response (DDR), cellular senescence was seen as a stable arrest of the cell cycle. This existed within a Canonical Pathway that typically included telomere shortening, oncogene activation, exposure to radiation or damage to the DNA due to environmental hazards, and activation of the ATM/ATR pathway that stabilizes p53 protein and induces p21-CIP1 and/or p16-INK4a). This consequently leads to a permanent arrest in cell proliferation combined with a pro-inflammatory secretory phenotype (SASP) indicative of senescence. It is now becoming apparent that not all senescent states demonstrated to-date were initially created by direct nuclear damage to DNA. A separate state of senescence has been identified as Mitochondrial Dysfunction-Associated Senescence (MiDAS) that occurs mainly due to mitochondrial dysfunction and metabolic problems as opposed to classic genotoxins [100].

Long-lasting mitochondrial dysfunction can cause MiDAS; this includes cellular impairment of oxidative phosphorylation (OXPHOS), reduced mitochondrial membrane potential, abnormal cristae structure, and overproduction of mitochondrial reactive oxygen species (ROS). Their defining upstream driver is metabolic imbalance, particularly a disruption of the cellular redox state, thereby allowing MiDAS to occur without sustained nuclear γH2AX foci formation and/or robust ATM/ATR activation. A distinctive feature of MiDAS features a low NAD+/NADH ratio that will hinder mitochondrial bioenergetics and change sirtuin activity which, in turn, leads to the destabilization of mitochondrial proteostasis and stress responses [92]. This reduction of the redox environment promotes energy-level sensors, such as AMP-activated protein kinase (AMPK), which has downstream effects that lead to p53 phosphorylation and, thus, p21-mediated cell cycle arrest independent of classical DNA-damage signaling.

Mitochondrial dysfunction is due to decreased mitophagy and mitochondrial biogenesis, which regulate mitochondrial quality. Furthermore, the buildup of dysfunctional mitochondria creates a persistent feed-forward loop of metabolic stress, involving AMPK activation and altered NAD+ metabolism, which further exacerbates the buildup of dysfunctional mitochondria. Research, using both computational and experimental approaches, demonstrated that the stabilization of a self-sustaining senescent state can be driven by mitochondrial dysfunction in the absence of significant nuclear DNA damage, thereby emphasizing the independence of mitochondrial signaling from nuclear signaling [84]. This distinguishes the MiDAS phenotype from senescence due to DDR, as in DDR, mitochondrial dysfunction is typically only a consequence of nuclear DNA damage.

One critical distinction is the identity of the Secretory Associated Phenotypic Alteration (SASP) profiles related to these two forms of senescence. Canonical senescence induced by DNA damage is associated with a strong proinflammatory SASP, primarily composed of NF-kB-mediated cytokines such as IL-1 alpha, IL-1 beta, IL-6, and other inflammatory mediators, which propagate the inflammatory signaling cascade and potentially induce paracrine senescence. The SASP profile generated via mitochondrial deprivation apoplexy and stress (MiDAS) is quite different: while there may be some inflammatory components, the amplification loop dependent on IL-1 is diminished, and the SASP profile reflects metabolic stress rather than ongoing DNA damage response (DDR) signaling. This change in the SASP profile will be important for tissue remodeling, stem cell niches, and systemic age-related decline, as signals associated with mitochondria (i.e., mitochondrial DNA fragments and mitochondrial-derived vesicles) will likely affect immune activation and inflammaging in a context-dependent manner [81].

From a physiological standpoint, MiDAS may be particularly relevant in tissues with high energy requirements and/or low regenerative capacity (e.g., skeletal muscle, neurons, cardiomyocytes). Age-related depletion of NAD⁺, impairment of mitophagy, and accumulation of mitochondrial DNA mutations create a permissive environment for mitochondrial-driven senescence, even in the absence of clear genotoxic stress. This perspective aligns with the current view that mitochondrial dysfunction contributes to systemic decline and operates as a primary driver rather than a secondary consequence [100]. The differences between MiDAS and canonical DDR-mediated senescence have important clinical implications; for example, interventions targeting DNA damage checkpoints to correct DNA damage-associated senescence are unlikely to eliminate or prevent mitochondrial-driven senescence. Evidence suggests that MiDAS may be more responsive to interventions designed to restore mitochondrial homeostasis (e.g., replenishing NAD⁺, modulating AMPK activity, enhancing mitophagy, and improving mitochondrial biogenesis) [92]. These interventions aim to correct the underlying metabolic dysfunction rather than simply suppress downstream inflammatory signaling.

In conclusion, canonical senescence induced by DNA damage is driven by nuclear genomic instability and prolonged activation of DDRs, whereas MiDAS is primarily caused by mitochondrial dysfunction, leading to energy stress and redox imbalance. Even though both result in cells being in a state of stable arrest in G0/G1 (capable of re-entering G1 phase) and exhibiting a secreted modified state, the upstream triggers for initiation of the two types of senescence, the metabolism of the cells, and the inflammatory profile of the resulting cell types differ significantly. Thus, classifying MiDAS as a mechanistically unique form of senescence enhances our understanding of how mitochondrial dysfunction contributes to cellular senescence, aging, and age-related disease, and provides further support for developing targeted therapeutic strategies that restore mitochondrial quality control and enhance metabolic resilience.

Antioxidants targeting mitochondria are a hot research topic for alleviating potential mitochondrial damage in both humans and animals. Mitoquinone mesylate (MitoQ) is one of those antioxidants that builds up in the mitochondrial matrix. Preclinical investigations suggest that MitoQ mitigates oxidative damage and preserves mitochondrial DNA integrity in models of metabolic and cardiovascular diseases. Pilot and small-scale human trials have demonstrated enhanced endothelial function and reduced oxidative stress biomarkers; however, larger-scale trials yielding more definitive results are forthcoming [101, 102]. An examination of antioxidants targeting mitochondria illustrates both the promise and the constraints of this strategy: while mitochondrial delivery may enhance efficacy, it also raises concerns about dosage, unintended redox perturbations, and context-dependent effects [103, 104].

There are many reasons a clinical study on mitochondria-targeted antioxidants is often difficult to interpret. One reason this could occur is due to a framework of mitohormesis. Reactive oxygen species (ROS) that are generated in the mitochondria are responsible for causing oxidative damage and also as critical mediators of cellular adaptation to stress (e.g., inducing mitochondrial biogenesis, antioxidant response) through their ability to act as a signal for certain types (e.g., ROS-induced mitochondrial biogenesis) of adaptive responses and pathways. Moderate increases in ROS produced by a cell may actually promote resilience (i.e., increased ability to withstand stress), while excessive ROS scavenging (which is the primary function of the vast majority of antioxidants currently marketed) will diminish the effect of these adaptive signaling pathways and impede the capacity for physiological changes [105, 106].

As such, the efficacy (and/or effectiveness) of any mitochondria-target antioxidant (e.g., MitoQ) is likely to be highly dependent on the timing, dosing and biological environment in which it is applied. For example, the application of targeted antioxidants in preclinical models of acute/severe oxidative stress typically results in improvements in mitochondrial function and restored redox balance, while in heterogeneous human populations, especially those not exhibiting significant oxidative pathology, they are likely to have little (or no) positive effect (i.e., suppressing ROS-mediated beneficial signaling) [102, 107]. Furthermore, differences in baseline redox state and mitochondrial membrane potential within age ranges, and tissue-specific antioxidant capacities further increase variability.

In light of this information, it is likely that the be highly time-dependent and/or disease-specific. Strategies that attempt to inhibit ROS production/accumulation over an extended period—for chronic ROS suppression—are likely to impede ROS’s beneficial properties and ultimately fail to produce a positive outcome when applied to a human population. Conversely, a strategy that allows fine-tuning of mitochondrial redox signaling or that combines antioxidant delivery with strategies to support mitochondrial generation and turnover is much more likely to preserve the adaptive stress response and limit maladaptive damage (the focus of this article).

Another option is the body’s built-in mechanisms for quality control. Urolithin A (UA), a metabolite produced by the gut microbiome, has shown promise in improving mitochondrial and muscle biomarkers in humans and extending the lifespan of test organisms. UA promotes mitophagy. More research is needed to determine its impact on larger functional outcomes; however, randomized trials have shown that it is safe and beneficial for muscle endurance and mitochondrial biomarkers. As a comprehensive framework for translation, these findings developed a hierarchy of conserved mitopha­gy inducers by using mitochondrial biomarkers to confirm their efficacy, preceded by experimentation using selected patient populations (i.e., age-related frailty; sarcopenia) [108, 109].

One way to translate this is to focus on receptor-mediated pathways, such as NIX, FUNDC1, BNIP3, and the PINK1-Parkin ubiquitin pathway. To facilitate the removal of damaged mitochondria, small molecules that enhance Parkin recruitment, TBK1 activation, or the function of autophagy adaptors may be employed. Researchers are trying gene therapy methods that bring back Parkin/PINK1 expression in tissues that don’t express it very well to treat mitochondrial myopathies and Parkinson’s disease.66 However, there is a risk involved in directly activating Parkin/PINK1 because too much ubiquitination can cause proteostatic stress. It is possible to find a happy medium between the two by administering the molecules to specific tissues for brief periods.

In many cases, replacing cleared organelles needs a mix of mitophagy, metabolic support, and stimulation of mitochondrial biogenesis. Agents that activate PGC-1α, such as AMPK activators (e.g., metformin, newer direct AMPK agonists), NAD⁺ boosters (e.g., NR, NMN), and sirtuin modulators, can increase mitochondrial mass and function, and synergize with mitophagy enhancers [87, 91]. Combination therapies that pair mitophagy induction (clearance) with biogenesis stimulation (replacement) — for example, UA plus exercise or NAD⁺ precursors — are biologically plausible and supported by preclinical synergy data, but require clinical trials designed to evaluate combination effects and timing [86, 110].

Senolytics and senomorphics are related to mitochondrial therapies because old cells produce mitochondria that don’t function properly, which causes long-term inflammation. To enhance mitochondrial healing, eliminating senescent cells is advantageous, as it reduces inflammation and mitochondrial DAMPs. More studies are needed to determine whether combining senolytics with mitochondria-targeted therapy is safe. However, early clinical trials have shown that senolytics alone may not be enough to promote tissue regeneration and may have metabolic and cognitive benefits in some groups [100, 111].

Mitochondrial dysfunction represents a critical pathway to the development of a pro-inflammatory environment, leading to increased levels of pro-inflammatory markers and ultimately increasing the risk of chronic inflammatory diseases. Mitochondrial dysfunction also activates innate immunity by interacting with cytochrome c oxidase via mitochondrial DNA (mtDNA). The abnormal export of mtDNA into the cytosol results from impaired mitochondrial quality control, increased mitochondrial membrane permeability, and thereby facilitates mtDNA release. The resulting pro-inflammatory response from the cytosolic detection of mtDNA occurs due to the recognition of mtDNA by cGMP-AMP synthase via the generation of cyclic-diphosphate-adenosine; this is a key component of the pathway leading to type I interferon activation and activation of the NF-signaling pathway [24, 112].

Activation of the NLRP3 inflammasome occurs as a consequence of multiple converging pathways mediated by mitochondrial dysfunction; these include increased production of mitochondrial reactive oxygen species (ROS), externalization of cardiolipin from the mitochondria membrane and ionic imbalance all of which lower the threshold for NLRP3 inflammasome assembly [113, 114] and thus promote activation of the inflammasome. It is also important to note that mitochondrial dynamics regulate the inflammatory workings, where excessive fragmentation of mitochondria promotes mtDNA release and ROS signaling, while balanced mitochondrial fusions inhibit NLRP3 inflammasome activation through preserving mitochondrial integrity [115, 116]. Thus, by examining the mechanisms by which mitochondrial stress mediates pro-inflammatory signaling, we can understand the relationship between mitochondrial dysfunction, mitochondrial dynamics, and the development of chronic, sterile inflammatory responses associated with Ageing.

Mitochondrial DNA provides two functions in cellular biology. In addition to passive damage-associated molecular patterns, when released from the mitochondria, it will enter the cytosol and act as a signaling molecule by activating the innate immune response via the cyclic GMP-AMP synthase (cGas) -stimulator of Interferon Genes (STING) pathway. Age-related mitochondrial stress—characterized by impaired mitophagy, increased membrane permeabilization, or nucleoid instability—promotes the aberrant release of mitochondrial DNA into the cytosol, where it activates cGAS–STING signaling and downstream inflammatory pathways [117, 118]. Importantly, this pathway is particularly relevant in advanced age, when normal responses to mitigate mtDNA exposure to the cytosol are progressively lost due to reduced mitochondrial turnover and lysosomal mitochondrial degradation. In the absence of effective means to limit the activation of the cGas-STING signaling pathway, continual low level activation of this signaling pathway leads to chronic sterile inflammation, and contributes to the sustained interferon tone and immune dysregulation seen in aged tissue [119, 120].

The dynamics of mitochondria also regulate inflammation, depending on the individual’s age. In older individuals, changes in their distribution between fused and fragmented forms can cause an increase in activation of the inflammasome (process by which immune cells become activated), by allowing for increased production of mitochondrial ROS and also producing an environment in which there is increased exposure of mitochondrial cardiolipin and increased susceptibility of the mito-inflammasome complex to interact [113–121]. On the other hand, elongated/overactive mitochondrial fusion states can facilitate better regulation of the mechanism underlying excessive inflammasome assembly under normal conditions, suggesting a role for mitochondrial shape in controlling the expression of immune signals. With aging comes a loss of the normal balance between mitochondrial structures, that are important for bioenergetic output and quality control, and this results in increased pro-inflammatory signals, which feeds back into the process of mitochondrial dysfunction, which contributes to deteriorating function within tissues as a result of changes to the local inflammatory environment caused from non-resolving state of inflammation, additional alterations to the interactions between immune and stromal cells and a decrease in the ability of stem cells to regenerate. Hence, abnormal mitochondrial structure and/or function are linked to systemic inflammation, resulting in a syndrome called “inflammaging” that occurs as a consequence of aging [25, 122, 123].

The primary challenges in translation are delivery, targeting, and biomarkers. MitoQ and peptide inhibitors of fission, like P110, are two small chemicals that work on mitochondria. They build up in different ways in different organs. There are concerns about the immunogenicity and long-term safety of AAV vectors for gene delivery, but they can deliver tissue-specific genes (e.g., TFAM, PGC-1α isoforms, and corrected MFN2/OPA1 alleles). To assess mitochondrial health, we need biomarkers such as high-resolution respirometry in muscle biopsies, mitochondrial DNA signatures in the blood, mitophagy reporter readouts (if possible), metabolomic panels, and validated imaging endpoints [124]. During the initial phases of a trial, it is crucial to focus on mechanistic endpoints rather than broader clinical outcomes.

Safety and contextual factors should inform clinical strategies. In tissues characterized by elevated mitochondrial turnover or where reactive oxygen species (ROS) serve as essential signaling molecules (e.g., in immunological responses), interventions that generally enhance mitophagy or ROS scavenging may prove detrimental. Short-term treatments, such as those administered during peri-ischemic windows or after injury, may be more effective and safer than long-term systemic management. We can enhance signal detection and the risk-benefit balance by stratifying patients based on the presence of evident mitochondrial dysfunction (as indicated by biomarkers or functional testing), pertinent genotypes, or disease states most likely to respond [97, 102].

### Therapeutic limitations, risks, and population-specific considerations

Although the idea of using mitochondrial-targeted treatments is becoming increasingly popular, there are still some concerns about the safety of these treatments and the risks associated with using them in older patients or those with chronic diseases. This is because mitochondrial pathways play an essential role in a number of processes within each cell, including how cells communicate through the immune system (immunosignaling), how cells die via apoptotic mechanisms (apoptosis), and how cells adapt to changing metabolic demands (metabolic adaptation). Abrupt or chronic changes made to mitochondrial activity could lead to the disruption of normal physiological processes within the body (physiological homeostasis) as well as affect other cellular pathways [2, 76]; for instance, excessive clearing of reactive oxygen species (ROS) could also prevent normal functioning of redox-dependent signaling and innate immunological responses, while chronic stimulation of mitophagic activity and/or mitochondrial biogenesis could cause depletion or disruption to cellular structures involved in cellular energy production (organelle depletion/metabolic imbalance) [18, 125].

Therapeutic responses can be further complicated by age-related changes. For example, older adults often have lower physiological reserves, differences in pharmacokinetics, higher levels of chronic inflammation, and higher rates of multiple chronic conditions. All of these can result in increased adverse effects and a decreased therapeutic effect of treatment [126]. Furthermore, even though short-term studies of NAD+ enhancing approaches typically report that they are generally well tolerated, there may be differences in the effects of NAD+enhancing approaches in patients with cancer, chronic inflammation, and/or increased DNA damage because NAD+ plays a key role in providing energy for PARP activity, DNA repair, and cell metabolism [127, 128]. Likewise, senolytic therapy has the potential to cause off-target toxicity, thrombocytopenia, and temporary organ dysfunction, especially when administered systemically to older adults with frailty [129].

Modulators targeting mitochondrial dynamics and mitophagy not only have difficulty with translation but can also chronically interfere with physiological cellular function and development by affecting normal cellular processes (such as fission) and cellular responses to stress (such as fusion). Therefore, clinically, prolonged or excessive fission activities and sustained high levels of fusion in a tissue are physiologically damaging, as both fission and fusion are necessary for normal biological functions and quality control. The activation of PINK1–Parkin signalling is also limited to a short time window, after which the development of cytotoxic and/or toxic mitochondrial proteins occurs, leading to toxicity in other unaffected cells [12]. Consequently, the aforementioned risks point to the need for time-dependent dosing strategies based on biomarker-guided, tissue-targeted approaches for the administration of therapeutic agents aimed at restoring mitochondrial homeostasis [130].

Taken together, all of these elements reinforce the notion that there will most likely not be a universal approach for applying mitochondrial medicine. Therefore, for any successful clinical translation of mitochondrial therapies, it will be necessary to stratify patients carefully regarding their short-term and long-term risks associated with mitochondrial therapies, implement a balanced combination of interventions for both replacement and clearance of damaged mitochondria, and undertake ongoing monitoring for safety in select populations (e.g., the elderly). All of these steps must be addressed if we want to successfully transfer basic science into clinical settings for treating patients with potential benefits of mitochondrial therapies for the aging process and age-related diseases.

Therapeutic interventions utilizing mitochondrial biology encompass antioxidant delivery (MitoQ and related MTAs), induction of mitophagy (urolithin A and emerging small molecules), metabolic support (NAD⁺ boosters, AMPK/sirtuin activators), cellular strategies (senolytics, gene therapy), and modulation of mitochondrial dynamics and quality-control pathways (PINK1-Parkin, BNIP3/NIX, OPA1/MFN regulation). For translational research to work, it needs to have a clear understanding of how things work (target engagement and pathway specificity), well-planned clinical trials based on biomarkers, careful removal and replacement of faulty mitochondria, and careful selection of patient groups. After several early human trials and proof-of-principle studies in model organisms, the field must now advance to larger, more strategically designed trials that combine molecular endpoints with significant clinical outcomes while ensuring rigorous safety [131, 132].

**Fig. 9:**
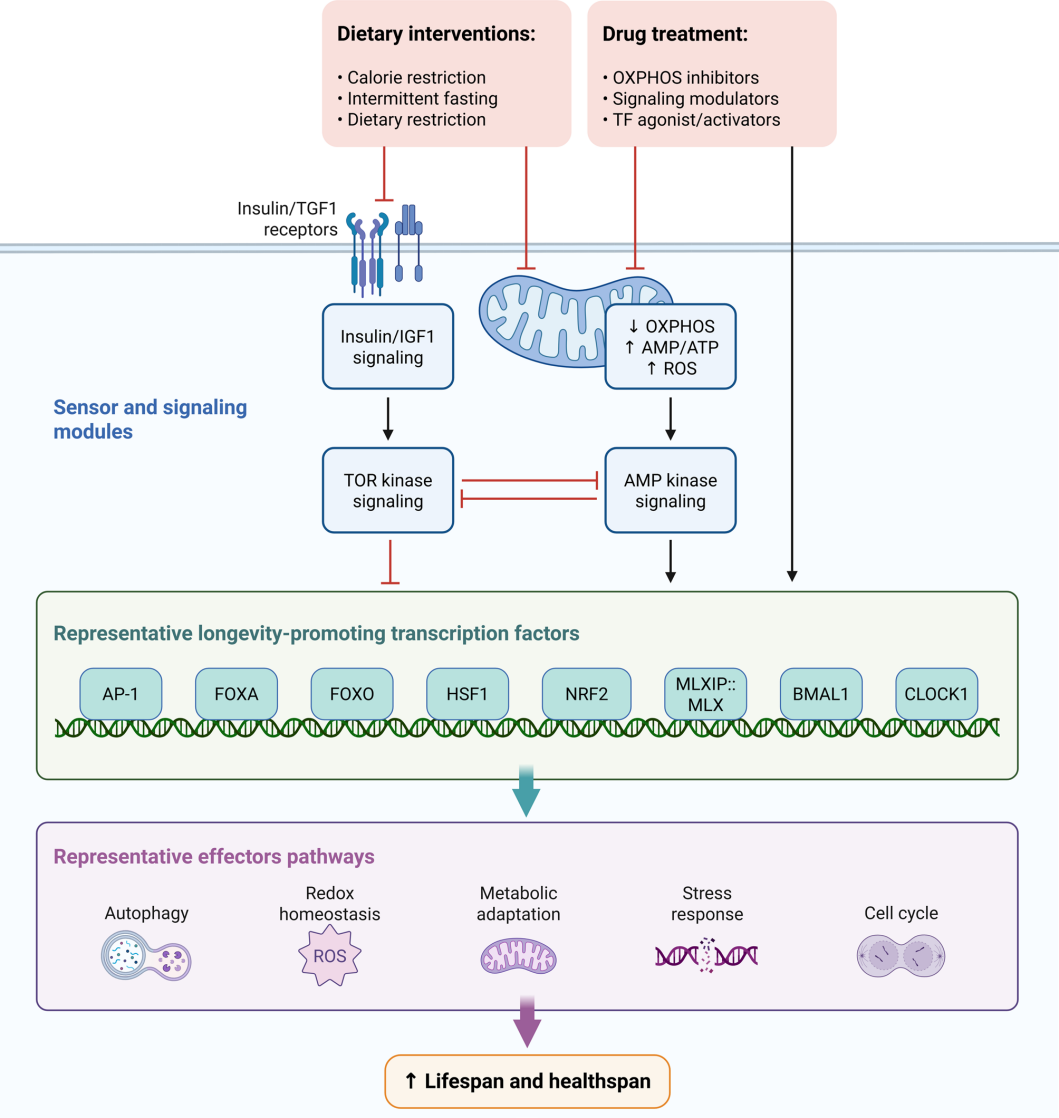
Shows that transcription factors that are highly conserved control how long an organism lives. This model depicts the pathways and responses to stress regulated by evolutionarily conserved transcription factors, including FOXO, HIF, NRF2, PGC-1α, and HSF1. These transcription factors are targets for geroprotective treatments because they combine signals from pathways that detect nutrition and react to stress—used with permission from Fischer et al., Physiology Review 2022. B. in Biomolecules 15, 860 (2025)

This model shows how evolutionarily conserved transcription factors, such as FOXO, HIF, NRF2, PGC-1α, and HSF1, regulate pathways and stress responses. These transcription factors get signals from pathways that sense nutrients and stress. They are excellent candidates for anti-aging treatments (Fig. 9).

The clinical translation of mitochondrial-targeted therapies has been hampered by a recurring disparity between their very strong efficacy in preclinical studies and the inconsistent and/or modest responses observed in human clinical trials. Several fundamental factors may be responsible for this discrepancy. Because mitochondrial redox (oxidation & reduction) signaling is highly context-dependent, excessive antioxidant activity can interfere with the normal roles of reactive oxygen species (ROS) in cellular physiological adaptation, a phenomenon known as the “antioxidant paradox.” [18, 105, 133]. This phenomenon may help to explain the observation that compounds such as MitoQ exhibit protective benefits in animal models that display a high degree of oxidative stress, but that provide no benefit (or at least a neutral benefit) to a heterogeneous population of people in which the redox imbalance is either mild or not as pronounced as in the animal model. Another factor contributing to the lack of concordance between preclinical efficacy and clinical effectiveness is the substantial variation in mitochondrial membrane potential, tissue-specific uptake, and intracellular distribution across animal and human studies [101, 134].

As far as population variability is concerned, the inter-individual differences in human populations with respect to mitochondrial genetic variation, baseline metabolism, inflammatory burden, and compensatory capacity may be significant factors in determining if a particular mitochondrial-targeted therapy restores the adaptive balance or exacerbates dysfunction in an individual patient. Thus, mitochondrial-targeted therapies, such as those involving NAD⁺ precursors or those that enhance mitophagy, may have a narrow biological window of opportunity for benefit and, when applied indiscriminately or long-term, may become ineffective or even maladaptive [14, 15].

These findings suggest that, in general, the lack of overall clinical success with these interventions is largely due to a failure to coordinate the combination of therapeutic mechanisms, target patient populations and the disease stage at which the interventions are being employed rather than to any inadequacy of the interventions. The success of future research will rely heavily on biomarkers for the selection of subjects for clinical trials, and on the development of and adherence to dosing strategies that take into account the context in which the intervention will be applied, as well as a move towards the precise modulation of mitochondrial function within a specific population rather than the implementation of a single, universal strategy for the modulation of mitochondrial function.

## Future directions and challenges

Several clinical trials are currently underway to investigate the potential development of novel drugs to improve mitochondrial health. Some of these compounds serve as antioxidants that target mitochondria, induce mitophagy, and boost NAD+, among other functions. This is good news, but it will be a long time before mitochondria-based medicines are used in everyday medical practice. Resolving these issues will enable the next stage of research in mitochondrial medicine to proceed.

In order for combination mitochondrial therapies to be successfully introduced into clinical use, the complexity of aging biology must be taken into account when designing clinical trials [135]. Therefore, biomarker-guided patient stratification based on mitochondrial function, inflammatory burden, metabolic status, or biological age, rather than chronological age should be prioritized in future clinical trials in order to provide more precise assessments of how well combinations of interventions respond to treatment [136, 137]. Furthermore, adaptive and factorial trial designs that allow for ongoing adjustments to dosage, sequence and/or combination of multiple higher-dose treatments are well-suited to this goal [138]. Since the aging process is inherently slow and heterogeneous, it is also necessary to use surrogate endpoints, such as improved energy production from mitochondria (bioenergetics), increased mitophagic efficiency (mitophagic flux), or increased activation of NAD+-dependent metabolic pathways (NAD+ metabolism) to supplement conventional clinical outcomes in developing feasible study durations [139]. There is also a need to identify potential drug-drug interactions, cumulative toxicity, and unique effects of combination strategies as the combination therapy model for aging interventions continues to evolve, particularly for older and multi-morbid patients. By integrating these fundamental principles into clinical studies, we will connect new mechanistic discoveries with evidence-based, clinically relevant interventions for the treatment of aging [16, 129].

### Improving delivery and bioavailability of mitochondrial therapeutics

Many drugs that target mitochondria don’t work well because they don’t enter the body effectively, break down poorly, or are not efficiently absorbed by mitochondria. Nicotinamide Mononucleotide (NMN) and Mitoquinone (mitochondria-targeted coenzyme Q10 derivative) are two examples of these mitochondrial-directed drugs. Several strategies are suggested to enhance the delivery of these components to mitochondria, including the development of new formulation technologies, nanocarrier conjugation, and the use of natural signals to target mitochondria. To facilitate the delivery of these drugs into mitochondria, several methods. Still, other challenges in delivering mitochondrial-targeted drugs remain, including issues with tissue selectivity and accumulation. Additionally, there is still a need for further investigation into precision targeting, which is crucial for drugs to target mitochondria in disease-relevant tissues while minimizing adverse effects.

Aging significantly impacts the major functions of mitochondria. Table 1 summarizes the cellular and physiological changes in these mitochondrial functions as they age.

**Table 1:** Major mitochondrial functions affected by aging and associated cellular and physiological changes

Mitochondrial Function	Aging-Related Cellular Changes	Associated Physiological Consequences
ATP Production (Oxidative Phosphorylation)	Reduced efficiency of electron transport chain complexes; decreased ATP synthesis	Decline in cellular energy availability; reduced tissue and organ function
Reactive Oxygen Species (ROS) Regulation	Increased mitochondrial ROS production; impaired antioxidant defenses	Accumulation of oxidative damage to proteins, lipids, and DNA
Mitochondrial DNA (mtDNA) Integrity	Accumulation of mtDNA mutations and deletions; reduced repair capacity	Impaired mitochondrial protein synthesis; progressive mitochondrial dysfunction
Mitochondrial Dynamics (Fusion and Fission)	Imbalance between fusion and fission processes; increased mitochondrial fragmentation	Disrupted mitochondrial network; reduced cellular adaptability to stress
Mitophagy and Quality Control	Decline in mitophagy efficiency; accumulation of dysfunctional mitochondria	Increased cellular stress and susceptibility to apoptosis
Regulation of Apoptosis	Altered membrane permeability; dysregulated release of pro-apoptotic factors	Enhanced cell loss contributing to tissue degeneration
Metabolic Signaling	Impaired mitochondrial–nuclear communication; altered metabolic signaling pathways	Dysregulation of metabolic homeostasis

### Understanding inter-tissue mitochondrial communication and systemic aging

The roles of mitochondria in maintaining healthy cellular function are enormous and extend beyond energy generation alone. Mitochondria act as communication hubs within cells, sending signals such as Mitochondrial peptides (e.g., MOTS-c), mitochondrial DNA fragments, and extracellular vesicle payloads between tissues, thereby affecting inflammation, metabolism, and aging. We don’t know precisely how mitochondria “talk” to each other in many parts of the body, like the immune system, muscles, and brain. To gain insight into the differential efficacy of certain medications on specific tissues or systems, it is essential to delineate these systemic interactions. This will help find new targets for therapy.

### Identifying reliable biomarkers of mitochondrial aging

One of the existing barriers to translating mitochondrial research into clinical practice is the lack of reliable biomarkers for assessing mitochondrial health. Ongoing methods used for evaluating mitochondrial functions include mitophagy reporter systems, metabolomic panels, high-resolution respirometry, and circulating cell-free mitochondrial DNA. Although these methods do provide helpful information, they are not standardized, sensitive, or specific. Validated mitochondrial multimodal biomarker panels encompassing molecular readouts, physiological endpoints, and functional imaging would be essential for enhancing understanding of the complex dynamics of mitochondrial aging.

### Addressing safety and long-term efficacy of mitochondrial interventions

Designing therapeutic strategies aimed at restoring mitochondrial functions and homeostasis should be taken with great caution. Targeting and manipulation of mitochondrial functions, including mitophagy, fission/fusion dynamics, and ROS signaling, can have deleterious cellular-damaging effects if not adequately regulated. For instance, excessive ROS scavenging or excessive mitophagy can both weaken the immune system and lead to mitochondrial depletion. Longitudinal safety monitoring must be integrated into both early clinical trials and preclinical research to attain a balance between systemic risks and efficacy.

### Integrating mitochondrial restoration strategies with other anti-aging approaches

Mitochondrial therapy techniques ought to be integrated as fundamental elements of a comprehensive therapeutic plan. Strategies aimed at modulating cellular nutrition sensing, reducing senescence burden, and promoting cellular regeneration—including the application of senolytics, stem cell therapies, and metabolic regulators (e.g., metformin and rapamycin)—can significantly influence mitochondrial health. To augment stem cell regenerative potential, eradicate senescent cells, and improve mitochondrial turnover, synergistic treatment strategies are essential. There is not yet an optimal combination of medicines that has the least harmful effects and the most advantages. This requires the logical formulation of integrated precision medicine approaches grounded in precise data, including genetic background, disease context, and biomarkers.

The future of mitochondrial treatment is quite promising. Research focused on identifying improved delivery mechanisms, mapping systemic communication pathways, discovering novel biomarkers, and conducting long-term safety evaluations is a critical stage toward the development of innovative mitochondria-targeted therapeutics. Combining mitochondrial restoration with other anti-aging treatments may also be a beneficial approach to enhance health and reduce the risk of age-related diseases. This could be achieved by employing a multidisciplinary approach that incorporates pharmacology, mitochondrial biology, systems medicine, and geoscience. This would lead to the creation of new compounds and the advancement of this field.

## Conclusion

Mitochondrial aging is best conceptualized as a decline in the level of information processing that results from the deterioration of systems across different levels of an organism, through the disruption of interactions among and between various components of their networks. Mitochondrial DNA (mtDNA) maintenance, mitochondrial dynamics, mitochondrial metabolism, and mitochondrial quality control (MQC) systems converge to impair mitochondria’s ability to integrate into cellular networks, leading to increased dysfunction throughout those networks. Future research and therapeutic approaches directed at restoring the adaptive capacity and coordinated mitochondrial network level(s) of integration should be considered to replace or supplement current approaches aimed at treating individual pathways on a stand-alone basis.

The importance of seeing mitochondria as part of an integrated system is that evaluations of interventions that target mitochondria should not be based solely on whether they improve isolated processes (e.g., biogenesis, antioxidant ability, or mitophagy). Future studies must identify the point at which particular processes shift from being adaptive to maladaptive and what the biological context is for those processes. The field faces several challenges, including: (1) defining the limits that separate compensation from failure for mitochondrial function; (2) developing biomarkers that reflect functional self-sufficiency instead of just damage that has occurred; and (3) determining how variation in mitochondrial genetics in different tissues may affect the trajectory for systemic aging [2, 16, 140]. Answering these challenges will require longitudinal, systems approaches that permit molecular, metabolic, and functional profiling to be integrated across all stages of life. The transition from cataloguing mitochondrial defects to studying their dynamic regulation will be essential for advancing mechanistic understanding and creating meaningful translational impact.

Two examples of new and groundbreaking medications that show how fixing mitochondria could help people live longer are metabolic regulators and mitophagy inducers. But there are still many challenges that need to be fixed. For clinical translation to be effective, we require a comprehensive understanding of how mitochondria communicate across tissues, improved administration and bioavailability, long-term safety, and validated measures of mitochondrial aging. If you combine it with stem cell treatments and senolytics, you can receive even more anti-aging benefits. Studying mitochondria can help us understand the mechanisms of aging and lead to new approaches for treating it. Mitochondrial research could revolutionize the way we combat age-related diseases and extend our lifespan by enhancing diagnostics, treatments, and diagnostic tools.

## Data Availability

The paper and its supplementary information contain all the data supporting this review article.
